# Infrared Characterization of Mono-Hydrogenated Phenanthrene Isomers (1‑, 2‑, 3‑, 4‑, and 9‑HC_14_H_10_) in Solid *para*-Hydrogen

**DOI:** 10.1021/acs.jpca.5c08413

**Published:** 2026-04-02

**Authors:** Jun-Ying Feng, Yuan-Pern Lee

**Affiliations:** † Department of Applied Chemistry and Institute of Molecular Science, 34914 National Yang Ming Chiao Tung University, Hsinchu 300093, Taiwan; ‡ Center for Emergent Functional Matter Science, 34914 National Yang Ming Chiao Tung University, Hsinchu 300093, Taiwan

## Abstract

Hydrogenated polycyclic aromatic hydrocarbons have been proposed both as potential carriers of the unidentified infrared (UIR) bands and as catalytic sites for H_2_ formation in astrophysical environments. We report the infrared (IR) spectra for five monohydrogenated phenanthrene isomers (1-, 2-, 3-, 4-, and 9-HC_14_H_10_), generated by electron bombardment of phenanthrene (C_14_H_10_) codeposited with *para*-hydrogen onto a cryogenic substrate. Distinct absorption signatures intensified during extended dark storage of the matrix, while subsequent irradiation at 423, 380, 315, and 223 nm produced characteristic photochemical responses, enabling classification into five distinct groups. Assignments to individual isomers were supported by comparison with scaled harmonic vibrational wavenumbers and IR intensities calculated at the B3LYP/6-311++G­(d,p) level of theory. Hydrogen addition was observed at all accessible nonbridging carbon sites. The resulting spectra exhibit intense features in the 11.5–14.5 μm region, indicating that these species are unlikely to represent major contributors to the UIR emission bands.

## Graphical abstract

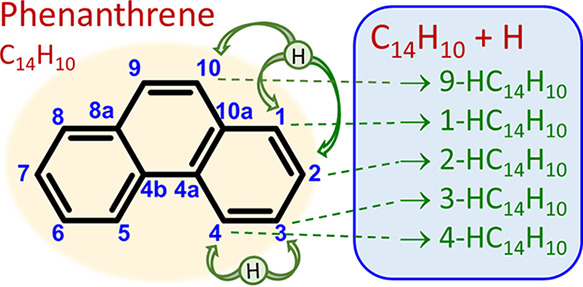

## 1 Introduction

Hydrogenated polycyclic aromatic hydrocarbons (HPAHs) are polycyclic aromatic hydrocarbons (PAHs) that incorporate additional hydrogen atoms into their carbon skeletons, partially saturating the aromatic rings and introducing aliphatic C–H bonds.^1^ In astrochemistry, these species serve as a bridge between the chemical and spectral properties of aromatic and aliphatic carbonaceous materials in space.^2^ PAHs are abundant in the interstellar medium (ISM) and have long been proposed as carriers of the unidentified infrared (UIR) emission bands; however, their infrared spectral signatures are strongly modulated by the degree of hydrogenation.^1^ The formation and destruction of HPAH are governed by the competition between hydrogen addition and photodissociation, processes that depend sensitively on local physical conditions, such as ultraviolet (UV) radiation flux and atomic hydrogen density.^3^ In dense or shielded regions, PAHs tend to become superhydrogenated, whereas in highly irradiated environments, dehydrogenation dominates, especially for PAHs of small-to-medium sizes. These hydrogenation dynamics not only modify the vibrational and electronic properties of PAHs but also play a pivotal role in astrochemical networks. HPAHs have been proposed as efficient catalysts for molecular hydrogen (H_2_) formation via abstraction reactions, thereby contributing to the molecular inventory of the ISM.^2,4^ Furthermore, the extent of hydrogenation influences the chemical resilience and evolutionary pathways of PAH, linking them to the broader life cycle of carbonaceous matter, including fullerenes and amorphous carbon grains.^2^ A comprehensive understanding of the structure, stability, and reactivity of HPAH is therefore essential for interpreting interstellar infrared spectra, modeling dust evolution, and tracing the carbon budget across diverse astrophysical environments.

The collective efforts of many researchers have been instrumental in understanding the spectroscopy of HPAH and their role in the ISM. For example, Allamandola and co-workers provided the foundational infrared evidence that extra hydrogen atoms give rise to the characteristic ∼3.4 μm aliphatic CH-stretching band, which often accompanies the 3.3 μm aromatic feature in astronomical observations.^5^ Joblin and co-workers further refined this by demonstrating that the 3.4/3.3 μm intensity ratio serves as a diagnostic tool for the UV radiation environments.^6^ Oomens and co-workers used gas-phase UV–IR double resonance spectroscopy, employing a two-color resonance-enhanced multiphoton ionization (REMPI) scheme, to show that hydrogenation might well account for the observed anomalously strong 3-μm “plateau” features of carbon-rich and star-forming regions.^7^ Complementing these gas-phase studies, Bouwman and co-workers reported that vacuum UV (VUV) irradiation of CO ice containing pyrene and water leads to the formation of hydrogenated pyrene, illustrating that PAH hydrogenation is an efficient process under cryogenic conditions relevant to interstellar environments.^8^

Phenanthrene (C_14_H_10_) consists of three fused benzene rings; its structure and carbon numbering are shown in Figure 1. Owing to molecular symmetry, positions 8, 7, 6, 5, 4b, 8a, and 9 are equivalent to positions 1, 2, 3, 4, 4a, 10a, and 10, respectively. Thus, seven distinct sites are feasible for protonation or hydrogenation, conventionally designated as 1, 2, 3, 4, 4a, 8a, and 9. Cané et al. recorded the IR absorption spectra of gaseous C_14_H_10_ and its fully deuterated analogue C_14_D_10_.^9^ Hudgins and Sandford reported the IR absorption spectra of C_14_H_10_ isolated in an Ar matrix, with observed vibrational wavenumbers in good agreement with scaled harmonic values predicted using the B3LYP/6-31G* method.^10,11^ With respect to the hydrogenation of C_14_H_10_, Thomas et al. investigated the reaction pathway leading to 1,4-dihydrophenanthrene via cross-beam reactions of the 1-naphthyl radical (C_10_H_7_^•^) with 1,3-butadiene (C_4_H_6_).^12^ Durland and Adkins successfully synthesized 9,10-dihydrophenanthrene, 1,2,3,4,5,6,7,8-octa-hydrophenanthrene, and tetradeca-hydro-phenanthrene from C_14_H_10_ using various metal catalysts.^13^ Yang et al. further demonstrated the hydrogen addition and abstraction reactions of 9,10-dihydrophenanthrene employing a CoMo/Al_2_O_3_ catalyst.^14^ Despite these advances, the identification of isomers of monohydrogenated phenanthrene (HC_14_H_10_), including their IR absorption spectra, has not yet been reported.

**Figure 1:**
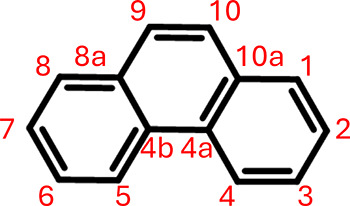
Carbon numbers of phenanthrene. Numbers 9, 8a, 8, 7, 6, 5, and 4b are equivalent to 10, 10a, 1, 2, 3, 4, and 4a, respectively, owing to molecular symmetry.

In our previous study,^15^ we reported the IR spectra of four isomers of protonated phenanthrene (1-, 3-, 4-, and 9-H^+^C_14_H_10_) isolated in solid *para*-hydrogen (*p*-H_2_) matrices, generated via electron bombardment of a mixture of C_14_H_10_ and *p*-H_2_ during deposition. In those experiments, monohydrogenated phenanthrene isomers (HC_14_H_10_) were also formed. Here, we present the IR spectral assignments of five isomers of HC_14_H_10_ (1-, 2-, 3-, 4-, and 9-HC_14_H_10_) in solid *p*-H_2_. All feasible hydrogenated C_14_H_10_ isomers, except those involving hydrogenation at the bridging carbons (4a and 8a), have been observed.

## 2 Methods

The experimental procedures for *para*-hydrogen matrix-isolation IR absorption and for the production of hydrogenated phenanthrene are identical to those used in our previous experiments for protonated phenanthrene.^15–18^ Hence, only a brief description is given here. IR absorption in the range of 500–3200 cm^–1^ was recorded after each experimental step using a Fourier-transform infrared (FTIR) spectrometer at a resolution of 0.25 cm^–1^. *p*-H_2_ was converted from normal hydrogen by using a hydrated iron­(III) oxide catalyst at 13.3 K.^18,19^ The *o*-H_2_ impurity after conversion was estimated to be less than 1200 ppm (parts per million) using the method of Tam and Fajardo.^20^

The matrix was prepared by passing *p*-H_2_ over solid C_14_H_10_ at 298 K, followed by deposition onto the substrate at 3.2 K under an electron bombardment for 8 h. The electron gun was operated at 450 eV with a current of 15 μA. Hydrogenated phenanthrene, along with protonated phenanthrene, was produced after deposition, and the former increased in intensity after the matrix was kept in darkness for 16 h. Secondary irradiation was performed at 423, 380, 315, and 223 nm (OPO laser, 10–20 mW at 10 Hz) to classify observed bands into various groups based on their distinct photolytic responses. Phenanthrene (C_14_H_10_, Chem. Service, 98.7%) was used as received.

Theoretical calculations were carried out using the Gaussian 16 program.^21^ The B3LYP hybrid functional^22,23^ with the 6-311++G­(d,p) basis set^24^ was used to optimize the structures and predict vibrational wavenumbers of all species investigated in this work. The same scaling equations as those employed for protonated phenanthrene: *y* = 0.9548*x* + 27.9 cm^–1^ for *x* > 2000 cm^–1^ and *y* = 0.9804*x* + 2.33 cm^–1^ for *x* < 2000 cm^–1^, in which *y* is the scaled wavenumber and *x* is the predicted harmonic vibrational wavenumber,^15^ were used for hydrogenated phenanthrene. Anharmonic vibrational analysis was performed using the VPT2 (second-order vibrational perturbation theory) method.^25^ Single-point energies were calculated with CCSD­(T), the coupled-cluster method including single, double, and perturbative triple excitations,^26^ according to geometries optimized with the B3LYP/6-311++G­(d,p) method. All energies were corrected for harmonic ZPVE, the zero-point vibrational energy, computed with the B3LYP/6-311++G­(d,p) method.

## 3 Results

### 3.1 Computational Results

The optimized geometries of seven possible isomers of monohydrogenated phenanthrene (HC_14_H_10_) are presented in Figure S1. The Cartesian coordinates for each species are given in Table S1. The ZPVE-corrected CCSD­(T) energies were also listed. The carbon frameworks of 4a- and 8a-HC_14_H_10_ are nonplanar (with C_1_ symmetry), whereas all other isomers remain planar (with *C*_*s*_ symmetry).

Table 1 summarizes the relative energies of the HC_14_H_10_ isomers with respect to 9-HC_14_H_10_, together with the hydrogen migration barriers for each isomer (Figure 2). Formation barriers for all isomers of HC_14_H_10_ derived from the addition of hydrogen to C_14_H_10_ are below 32 kJ mol^–1^, except for 4a- and 8a-HC_14_H_10_. Isomers 1-, 2-, 3-, and 4-HC_14_H_10_ exhibit relative energies within 25 kJ mol^–1^ of 9-HC_14_H_10_, whereas 4a- and 8a-HC_14_H_10_ are significantly less stable, exceeding the energy of 9-HC_14_H_10_ by 60 and 72 kJ mol^–1^, respectively. The barriers for hydrogen migration between adjacent sites generally fall in the range of 145–187 kJ mol^–1^.

**Figure 2:**
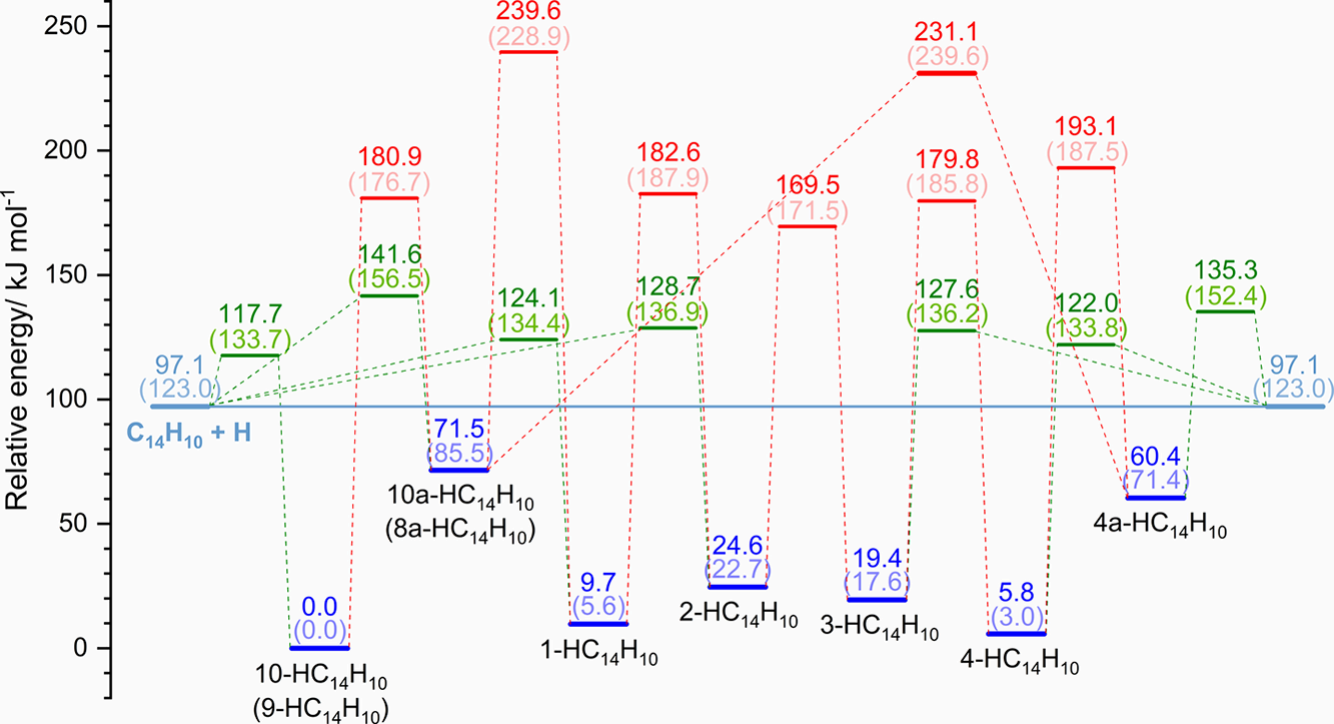
Potential energy scheme for the formation and hydrogen migration of various isomers of HC_14_H_10_. All energies are relative to the energy of 9-HC_14_H_10_. Energies of isomers of HC_14_H_10_, calculated with the CCSD­(T)/6-311++G­(d,p)//B3LYP/6-311++G­(d,p) method and corrected for zero-point vibrational energies (ZPVE) according to harmonic vibrational wavenumbers calculated with the B3LYP/6-311++G­(d,p) method, are indicated in blue. Those of transition states for isomerization are in red, and those for formation from H + C_14_H_10_ are in green. ZPVE-corrected B3LYP/6-311++G­(d,p) energies are listed in parentheses for comparison.

**Table 1:** Relative Energies (kJ mol^–1^) of Isomers of HC_14_H_10_ and Transition States (TS) for Formation and Hydrogen Migration Calculated with the CCSD­(T)/6-311++G­(d,p)//B3LYP/6-311++G­(d,p) Method

			H transfer		
species	relative energy^a^/kJ mol^–1^	barrier for formation^b^/kJ mol^–1^	transfer	TS energy^c^/kJ mol^–1^	barrier/kJ mol^–1^
9-HC_14_H_10_	0.0	20.6	10 (9)^d^ → 10a	180.9	180.9
8a-HC_14_H_10_	71.5	44.5	10a (8a)^e^ → 1	239.6	168.1
1-HC_14_H_10_	9.7	27.0	1 → 2	182.6	172.9
2-HC_14_H_10_	24.6	31.6	2 → 3	169.5	144.9
3-HC_14_H_10_	19.4	30.5	3 → 4	179.8	160.4
4-HC_14_H_10_	5.8	24.9	4 → 4a	193.1	187.3
4a-HC_14_H_10_	60.4	38.2	4a → 10a	231.1	170.7
H + C_14_H_10_	97.1				

a Zero-point vibrational energies (ZPVE) were corrected according to harmonic vibrational wavenumbers calculated with the B3LYP/6-311++G­(d,p) method.

b The barrier for the formation of isomers of HC_14_H_10_ is relative to the energy of H + C_14_H_10_.

c Transition-structure (TS) energy for H transfer is relative to the energy of 9-HC_14_H_10_.

d Sites 9 and 10 are equivalent. Conventionally, the preferred designation is 9-HC_14_H_10_.

e Sites 8a and 10a are equivalent. Conventionally, the preferred designation is 8a-HC_14_H_10_.

The scaled harmonic vibrational wavenumbers and IR intensities of 9-, 1-, 2-, 3-, 4-, 4a-, and 8a-HC_14_H_10_ are summarized in Tables S2–S4, with their IR stick spectra compared to that of C_14_H_10_ in Figure 3. Anharmonic vibrational wavenumbers and IR intensities for 9-, 1-, 2-, 3-, and 4-HC_14_H_10_ are also provided in Tables S2 and S3. Hydrogenation activates vibrational modes in the region of 650–850 cm^–1^, while characteristic CH_2_-stretching modes of the 9-, 1-, 2-, 3-, and 4-HC_14_H_10_ isomers appear in the region of 2800–2850 cm^–1^. Anharmonic calculations further predict numerous combination bands in the CH-stretching region arising from Fermi resonance; however, these bands are strongly overlapped with those of the fundamental bands.

**Figure 3:**
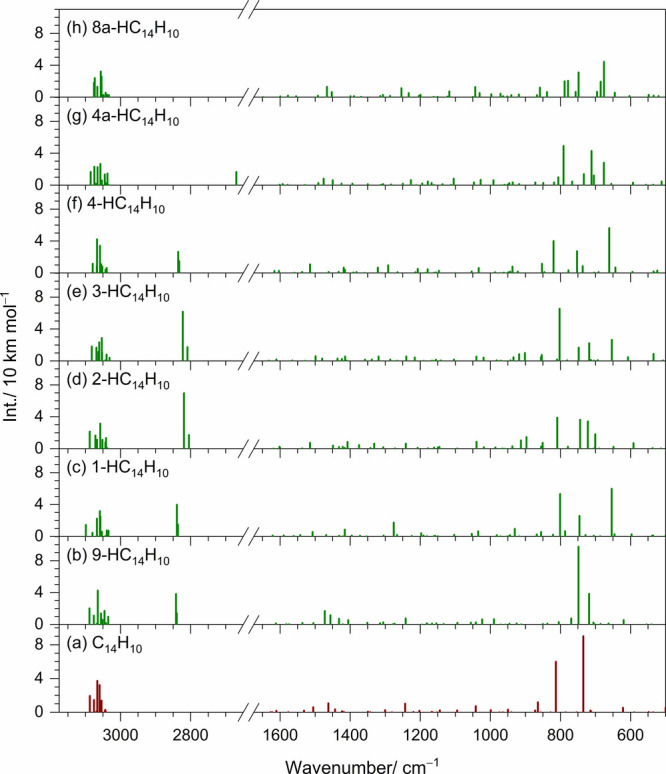
Computed stick spectra of C_14_H_10_ and isomers of HC_14_H_10_. (a) C_14_H_10_, (b) 9-HC_14_H_10_, (c) 1-HC_14_H_10_, (d) 2-HC_14_H_10_, (e) 3-HC_14_H_10_, (f) 4-HC_14_H_10_, (g) 4a-HC_14_H_10_, and (h) 8a-HC_14_H_10_. The spectra were based on scaled harmonic vibrational wavenumbers and IR intensities calculated with the B3LYP/6-311++G­(d,p) method.

### 3.2 IR Spectra of Mono-Hydrogenated Phenanthrene

The IR spectrum of C_14_H_10_ isolated in solid *p*-H_2_ has been discussed in our previous study.^15^ The observed spectrum agrees satisfactorily with theoretical predictions and with the spectrum of C_14_H_10_ isolated in solid Ar.^10^ Upon electron bombardment of a mixture of C_14_H_10_ and *p-*H_2_ during matrix deposition, both H^+^C_14_H_10_ and HC_14_H_10_ were produced. After prolonged maintenance of the matrix, H^+^C_14_H_10_ reacted with electrons trapped in the matrix to form HC_14_H_10_; some HC_14_H_10_ were also produced from the reaction of H + C_14_H_10_. Consequently, the IR features of H^+^C_14_H_10_ and HC_14_H_10_ could be readily distinguished by their decrease (H^+^C_14_H_10_) or increase (HC_14_H_10_), respectively, after dark storage of the matrix. Secondary photoirradiation further distinguished various isomers. Four isomers of the protonated phenanthrene, 1-, 3-, 4-, and 9-H^+^C_14_H_10_, have been discussed in our previous paper.^15^ In this work, we focus on the spectral assignments of five isomers of monohydrogenated phenanthrene.

In general, H^+^PAH exhibits IR features characterized by intense bands in the region of 1150–1600 cm^–1^ (mainly due to CH in-plane bending and CC-stretching modes) and weak absorption bands in the 600–900 cm^–1^ region (primarily due to CH out-of-plane bending modes). In contrast, HPAH displays intense bands in the 600–900 cm^–1^ range and weak features in the 1150–1600 cm^–1^ range. Consequently, interference from H^+^C_14_H_10_ for the key bands of HC_14_H_10_ is insignificant.

Figure 4a shows the IR spectrum of C_14_H_10_ in solid *p-*H_2_ in the region of 710–830 cm^–1^ for comparison. Figure 4b displays the corresponding IR spectra of a C_14_H_10_/*p*-H_2_ matrix after deposition with concurrent electron bombardment for 8 h in a separate experiment. Figure 4c shows the difference spectrum of the matrix after 16 h of dark maintenance. During this step, remaining electrons trapped in the *p*-H_2_ matrix neutralized protonated phenanthrene to form hydrogenated phenanthrene, and remnant hydrogen atoms can also react with phenanthrene to form hydrogenated phenanthrene, as indicated by the increase in intensity of some features by 116–159%. Subsequently, irradiations at 423, 380, 315, and 223 nm (laser energy of ∼1.5 mJ at 10 Hz, with a duration of 20 min for each wavelength) were carried out sequentially. The resulting difference spectra, shown in Figure 4d–g, were obtained by subtracting the spectrum recorded before laser irradiation from that recorded after irradiation. Figure S2 displays corresponding spectra covering an extended region of 575–1525 cm^–1^; additional difference spectra after prolonged maintenance of the matrix in darkness are also shown.

**Figure 4:**
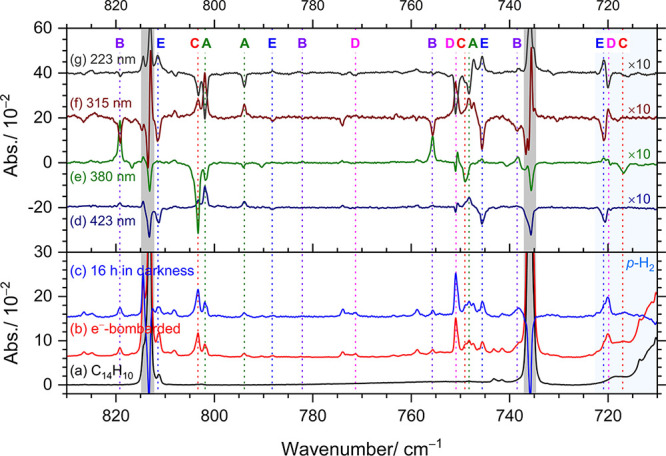
Representative infrared spectra of an electron-bombarded C_14_H_10_/*p*-H_2_ matrix after each experimental step. (a) C_14_H_10_/*p*-H_2_ matrix without electron bombardment. (b) Electron-bombarded C_14_H_10_/*p*-H_2_ matrix. (c) Spectrum recorded after maintenance of the matrix in darkness for 16 h (with most bands of C_14_H_10_ subtracted). Difference spectra after secondary irradiation at 423 nm (d), 380 nm (e), 315 nm (f), and 223 nm (g); each irradiation step is 20 min. Lines in groups A, B, C, D, and E are denoted with color-coded labels and dotted lines. For clarity, some features due to protonated species^15^ are not labeled. Spectral regions subjected to interference from the intense absorption of C_14_H_10_ are shaded gray, and those from that of *p*-H_2_ are shaded light blue. Baselines were shifted for clarity.

Table 2 summarizes the estimated mixing ratios of various species during each experimental step, with percentage variations after each step listed in parentheses. The mixing ratio *X* was estimated according to Beer’s law,^18,27^

$$X=\frac{2.303\int_{\mathrm{band}}A\left(\mathrm{\nu ̃}\right)d\mathrm{\nu ̃}}{\varepsilon \left(\mathrm{cm}\;{\mathrm{mol}}^{-1}\right)l\left(\mathrm{cm}\right)}\times \left(23.16\;{\mathrm{cm}}^{3}\;{\mathrm{mol}}^{-1}\right)\times 10^{6}$$ 1

in which ∫_band_ *A*(υ̃)­dυ̃ is the integrated absorbance in cm^–1^, ε is the IR intensity from theoretical calculations, *l* is the optical path length, and 23.16 cm^3^ mol^–1^ is the molar volume of solid *p*-H_2_ at 4 K. The IR optical path length *l* was determined according to the rotational *U*_0_(0) absorption band near 1167 cm^–1^ of *p*-H_2_, as described by Fajardo.^28^ The integration ranges for bands of each species are provided in Table S5. Given the inherent uncertainties in the calculated IR intensities, the estimated mixing ratio may be subject to significant errors. To reduce this error, multiple absorption lines of the same species were used to derive an average mixing ratio.

**Table 2:** Estimated Mixing Ratios of Isomers of HC_14_H_10_ after Each Experimental Step

assignment (group)	deposition^a^/ppm	16 h in darkness/ppm^b^	423 nm/ppm^b^	380 nm/ppm^b^	315 nm/ppm^b^	223 nm/ppm^b^
1-HC_14_H_10_ (A)	1.24 ± 0.08	2.91 ± 0.07 (+135%)	3.37 ± 0.04 (+16%)	3.10 ± 0.07 (−8%)	4.02 ± 0.38 (+30%)	2.87 ± 0.11 (−29%)
4-HC_14_H_10_ (B)	0.93 ± 0.32	2.01 ± 0.17 (+116%)	2.18 ± 0.10 (+8%)	3.36 ± 0.06 (+54%)	2.57 ± 0.18 (−24%)	2.47 ± 0.04 (−4%)
3-HC_14_H_10_ (C)	2.04 ± 0.20	4.74 ± 0.12 (+132%)	4.74 ± 0.12 (+0%)	3.35 ± 0.38 (−29%)	3.20 ± 0.23 (−4%)	3.03 ± 0.24 (−5%)
9-HC_14_H_10_ (D)	1.79 ± 0.66	4.00 ± 0.14 (+123%)	4.00 ± 0.14 (+0%)	4.04 ± 0.06 (+1%)	4.36 ± 0.14 (+8%)	3.90 ± 0.04 (−11%)
2-HC_14_H_10_ (E)	2.00 ± 0.93	5.17 ± 0.52 (+159%)	4.25 ± 0.55 (−18%)	4.32 ± 0.11 (+2%)	3.01 ± 0.38 (−30%)	3.80 ± 0.63 (+26%)

a Mixing ratios after depositions with electron bombardment.

b The percentage variations of absorbance after each step are given in parentheses.

These features, which increased in intensity after maintenance in darkness, can be categorized into five groups, denoted as groups A–E in Figure 4 and Figure S2, based on their distinct responses to secondary irradiations. Typically, features of the parent also decreased in darkness due to its reaction with H atoms, which interfered with the observed intensities of new features; therefore, the apparent relative intensities of new features may not be reliable. Features in group A, indicated with green dashed lines and label A, increased by ∼0.5 ppm (16%) and ∼0.9 ppm (30%) on irradiation at 423 and 315 nm, respectively, but decreased by ∼0.3 ppm (−8%) and ∼1.2 ppm (−29%) at 380 and 223 nm, respectively; percentage variations after each step are listed parenthetically. Features in group B (violet) increased by ∼0.2 ppm (8%) and ∼1.2 ppm (54%) on irradiation at 423 and 380 nm, respectively, but decreased by ∼0.8 ppm (−24%) and ∼0.1 ppm (−4%) at 315 and 223 nm, respectively. Features in group C (red) remained nearly unchanged on irradiation at 423 nm but decreased by ∼1.4 ppm (−29%), ∼0.2 ppm (−4%), and ∼0.2 ppm (−5%) at 380, 315, and 223 nm, respectively. Features in group D (pink) remained nearly the same on irradiation at 423 and 380 nm, increased by ∼0.3 ppm (8%) at 315 nm, but decreased by ∼0.5 ppm (−11%) at 223 nm. Features in group E (blue) remained nearly unchanged on irradiation at 380 nm, increased by ∼0.8 ppm (26%) at 223 nm, but decreased by ∼0.9 ppm (−18%) and ∼1.3 ppm (−30%) at 423 and 315 nm, respectively. Table 2 summarizes these results.

Unlike H^+^C_14_H_10_, isomers of HC_14_H_10_ are predicted to have relatively intense CH-stretching bands (Figure 3). However, identifying HC_14_H_10_ features in the CH-stretching region is highly challenging, as C_14_H_10_ also exhibits intense CH-stretching bands (Figure S3a) above 3000 cm^–1^. Hence, only the characteristic CH_2_-stretching bands of HC_14_H_10_, predicted in the region of 2800–2850 cm^–1^, can be reliably identified. Two bands near 2824.3 and 2794.5 cm^–1^, which decreased significantly upon irradiation at 380 nm, were assigned to group C. Two features near 2818.9 and 2789.3 cm^–1^, which decreased at 423 and 315 nm and increased at 223 nm, were assigned to group E. Features near 2833.8 and 2814.6 cm^–1^ were identified as group A, showing decreases at 380 and 223 nm and increases at 423 and 315 nm. A band near 2810.8 cm^–1^, growing significantly at 380 nm but decreasing at 315 nm, was tentatively assigned to group B. As discussed in Sections 4.1 and 4.2, these features in groups A–E are assigned to 1-, 4-, 3-, 9-, and 2-HC_14_H_10_, respectively.

## 4 Discussion

### 4.1 Assignments of Features in Group B to 4-HC_14_H_10_ and Group C to 3-HC_14_H_10_

Spectral features in groups B and C in the region 700–865 cm^–1^ are displayed in Figure 5 and compared with predicted spectra of five low-lying isomers of HC_14_H_10_. These stick spectra are derived from scaled harmonic vibrational wavenumbers and IR intensities predicted by the B3LYP/6-311++G­(d,p) method. The spectrum measured after maintaining the matrix in darkness is shown in Figure 5c. The difference spectrum after irradiation at 380 nm is presented in Figure 5b. Features of groups B and C are indicated with violet and red lines, respectively, along with their corresponding labels. Corresponding spectra for groups B and C in the region 575–1525 cm^–1^ are presented in Figures S4 and S5, respectively. In these figures, stick spectra of two additional high-energy isomers of HC_14_H_10_, 4a-HC_14_H_10_ and 8a-HC_14_H_10_, are included for comparison.

**Figure 5:**
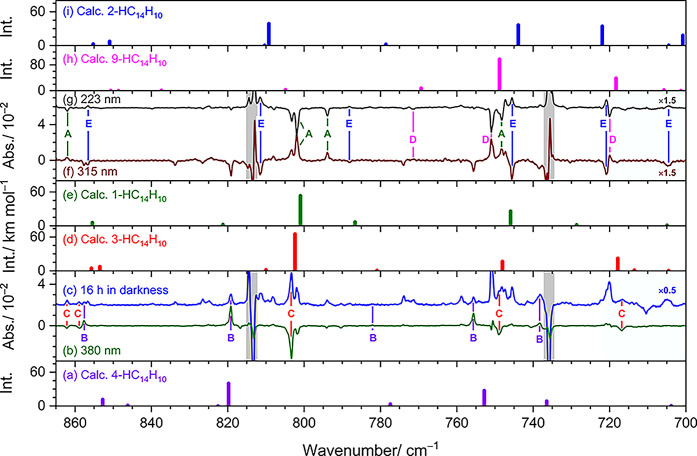
Comparison of bands in groups A, B, C, D, and E in the region 700–865 cm^–1^ with stick spectra of 4-HC_14_H_10_ (a), 3-HC_14_H_10_ (d), 1-HC_14_H_10_ (e), 9-HC_14_H_10_ (h), and 2-HC_14_H_10_ (i) according to scaled harmonic vibrational wavenumbers and IR intensities calculated with the B3LYP/6-311++G­(d,p) method. Panels (b), (c), (f), and (g) are experimental spectra taken from Figure 4. (c) Spectrum measured after maintenance of the matrix in darkness for 16 h (with most bands of C_14_H_10_ subtracted). Difference spectra after secondary irradiation at 380 nm (b), 315 nm (f), and 223 nm (g). Bands in groups A, B, C, D, and E are indicated with color-coded labels and solid lines. Spectral regions subjected to interference from the intense absorption of C_14_H_10_ are shaded gray, and those from that of *p*-H_2_ are shaded light blue. Baselines were shifted for clarity.

The observed spectral pattern of bands in group B agrees best with those predicted for 4-HC_14_H_10_ in wavenumbers and relative intensities (Figure 5a and Figure S4g, in violet) but matches less satisfactorily with other isomers of HC_14_H_10_ (Figure S4). The three most intense features, recorded at 819.2, 755.6, and 660.9 cm^–1^, correspond closely to the predicted vibrational wavenumbers near 820, 753, and 661 cm^–1^ (all associated with out-of-plane CH-bending modes) for 4-HC_14_H_10_. Additionally, two medium-intensity features were observed at 738.2 and 857.6 cm^–1^, showing good agreement with those predicted near 737 and 853 cm^–1^ (both out-of-plane CH-bending modes). As discussed in Section 3.2, the characteristic CH_2_-stretching mode was tentatively assigned at 2810.8 cm^–1^ (Figure S3), which might be attributed to either 2833 cm^–1^ (antisymmetric CH_2_-stretching mode) or 2836 cm^–1^ (symmetric CH_2_-stretching mode). Furthermore, 12 additional weaker bands also agree with predictions, as summarized in Table 3. Table 3 lists all vibrational modes predicted with IR intensity >5 km mol^–1^; the total number of observed vibrational modes is 18. Except for those associated with CH-stretching, which were obscured by the intense absorption of C_14_H_10_, all vibrational modes of 4-HC_14_H_10_ listed in Table 3 were observed. The mean absolute deviation between observed wavenumbers and predicted wavenumbers (scaled harmonic) of 4-HC_14_H_10_ is 5.3 ± 7.0 cm^–1^. The most significant deviations are 24.7 and 21.7 cm^–1^ for ν_10_ and ν_47_, which are typical for the CH_2_-stretching modes of hydrogenated PAHs. The features in group B are hence assigned to 4-HC_14_H_10_.

**Table 3:** Comparison of Experimental Vibrational Wavenumbers and Relative IR Intensities of 4- and 3-HC_14_H_10_ with Those Predicted Using the B3YLP/6-311++G­(d,p) Method

4-HC_14_H_10_ (group B)						3-HC_14_H_10_ (group C)					
mode	sym.	calculation		*p*-H_2_		mode	sym.	calculation		*p*-H_2_	
ν_1_	a′	3079.5^a^	(12)^b^	^c^		ν_1_	a′	3081.9^a^	(18)^b^	^c^	
ν_2_	a′	3067.5	(9)	^c^		ν_2_	a′	3068.9	(17)	^c^	
ν_3_	a′	3067.1	(42)	^c^		ν_3_	a′	3062.8	(11)	^c^	
ν_4_	a′	3058.5	(34)	^c^		ν_4_	a′	3060.6	(23)	^c^	
ν_5_	a′	3055.3	(11)	^c^		ν_5_	a′	3053.5	(29)	^c^	
ν_6_	a′	3051.9	(8)	^c^		ν_6_	a′	3052.6	(11)	^c^	
ν_9_	a′	3039.4	(6)	^c^		ν_8_	a′	3040.0	(8)	^c^	
ν_10_	a′	2835.5	(27)	2810.8?	(30)^d^	ν_10_	a′	2822.4	(62)	2824.3	(17)^d^
ν_15_	a′	1514.7	(11)	1514.3	(17)	ν_15_	a′	1499.0	(6)	1497.4	(8)
ν_18_	a′	1418.5	(7)	1414.6	(10)	ν_16_	a′	1479.8	(3)	1487.4?	(3)
ν_19_	a′	1415.0	(4)	1402.2	(4)	ν_19_	a′	1414.7	(6)	^e^	
ν_23_	a′	1321.2	(7)	1320.4	(4)	ν_22_	a′	1338.3	(2)	1331.6	(2)
ν_24_	a′	1291.5	(10)	1292.4	(6)	ν_23_	a′	1318.9	(6)	1321.9?	(1)
ν_27_	a′	1207.1	(5)	1207.8	(7)	ν_26_	a′	1239.8	(6)	1240.5	(2)
ν_28_	a′	1179.1	(5)	1182.5	(5)	ν_33_	a′	1039.9	(6)	^c^	
ν_31_	a′	1146.8	(3)	1141.6	(4)	ν_34_	a′	1019.2	(4)	1024.8	(4)
ν_34_	a′	1033.2	(6)	1031.8	(4)	ν_36_	a′	901.5	(10)	908.3	(4)
ν_36_	a′	936.9	(8)	944.5	(9)	ν_37_	a′	853.5	(8)	858.6	(6)
ν_47_	a″	2832.5	(15)	2810.8?	(30)	ν_41_	a′	607.4	(5)	604.2	(5)
ν_54_	a″	852.8	(12)	857.6	(26)	ν_47_	a″	2809.3	(17)	2794.5	(20)
ν_55_	a″	819.8	(40)	819.2	(100)	ν_52_	a″	934.0	(5)	930.2	(4)
ν_56_	a″	777.4	(3)	782.1	(4)	ν_53_	a″	917.7	(9)	911.9	(11)
ν_57_	a″	752.8	(27)	755.6	(63)	ν_54_	a″	855.7	(5)	862.0	(7)
ν_58_	a″	736.5	(9)	738.2	(23)	ν_55_	a″	802.4	(65)	803.3	(100)
ν_59_	a″	660.8	(56)	660.9	(57)	ν_57_	a″	748.1	(17)	749.0	(27)
ν_60_	a″	643.3	(7)	640.0	(7)	ν_58_	a″	717.8	(22)	716.8	(15)
						ν_60_	a″	653.3	(27)	651.1	(15)
						ν_61_	a″	534.4	(9)		

a Harmonic vibrational wavenumbers are scaled according to 0.9548*x* + 27.9 for wavenumbers >2000 cm^–1^ and 0.9804*x* + 2.3 for wavenumbers <2000 cm^–1^, in which *x* is the harmonic vibrational wavenumbers.

b Harmonic IR intensities (in km mol^–1^) are listed in parentheses. Only those with intensities >5 km mol^–1^ are listed.

c Obscured by C_14_H_10_.

d Percentage integrated intensities of each isomer are relative to the most intense band.

e Obscured by group B.

The predicted stick spectrum based on the anharmonic calculations of 4-HC_14_H_10_ (Figure S4h) shows poorer agreement than the scaled harmonic calculations. For example, anharmonic vibrational calculations yield values of 2768 and 2778 cm^–1^ for the CH_2_-stretching modes, which deviate by 43 and 33 cm^–1^ from the observed wavenumber of 2810.8 cm^–1^. No prominent combination or overtone bands were predicted for 4-HC_14_H_10_ (Table S3). The mean absolute deviation between observed wavenumbers and the anharmonic vibrational wavenumbers of 4-HC_14_H_10_ is 8.8 ± 11.2 cm^–1^.

The relative IR intensities and vibrational wavenumbers of group C agree satisfactorily with scaled harmonic vibrational predictions for 3-HC_14_H_10_ (Figure 5d and Figure S5f, in red) but show less agreement with other HC_14_H_10_ isomers (Figure S5). The most intense feature, observed at 803.3 cm^–1^, corresponds closely to the scaled harmonic vibrational wavenumber of an out-of-plane CH-bending mode predicted at 802 cm^–1^ for 3-HC_14_H_10_. A medium-intensity feature at 749.0 cm^–1^ also matches the predicted value near 748 cm^–1^ (also in an out-of-plane CH-bending mode). For the symmetric CH_2_-stretching mode, characteristic of HC_14_H_10_, two bands at 2794.5 and 2824.3 cm^–1^ in group C were observed (Figure S3), with intensities decreasing significantly upon irradiation at 380 nm. These wavenumbers are consistent with scaled harmonic vibrational wavenumbers at 2809 (antisymmetric CH_2_-stretching mode, ν_47_) and 2822 (symmetric CH_2_-stretching mode, ν_10_) cm^–1^, or a weaker overtone band (2ν_19_) near 2820 cm^–1^ (Table S3). Fourteen additional weaker bands are assigned to group C, all in good agreement with predictions for 3-HC_14_H_10_. As shown in Table 3, which summarizes all vibrational modes predicted to have IR intensity >5 km mol^–1^, 18 bands were identified for 3-HC_14_H_10_. All listed modes were observed, except for the CH-stretching modes and two predicted near 1040 (ν_33_) and 1415 (ν_19_) cm^–1^, which were interfered by absorption of C_14_H_10_ at 1040.7 cm^–1^ and 4-HC_14_H_10_ (group B) at 1414.6 cm^–1^, respectively. Thus, the bands in group C are assigned to 3-HC_14_H_10_. The average deviation (absolute values) between the observed and predicted vibrational wavenumbers (scaled harmonic) for 3-HC_14_H_10_ is 4.3 ± 3.5 cm^–1^, with the largest deviation of 15 cm^–1^ for ν_47_. The predicted stick spectrum based on anharmonic calculations of 3-HC_14_H_10_ (Figure S5g) also shows poorer agreement than the scaled harmonic calculations, with a maximal deviation of 47 cm^–1^. Even though anharmonic calculations improve over harmonic calculations, scaled harmonic calculations typically provide better agreement with the experiments. No prominent combination or overtone bands were predicted for 3-HC_14_H_10_ except for two in the region 3050–3090 cm^–1^ (Table S3). The mean absolute deviation between the observed and anharmonic vibrational wavenumbers of 3-HC_14_H_10_ is 12.7 ± 18.9 cm^–1^.

### 4.2 Assignment of Features in Groups A to 1-HC_14_H_10_, D to 9-HC_14_H_10_, and E to 2-HC_14_H_10_

The experimental spectra of features in groups A, D, and E in the region 700–865 cm^–1^ are compared in Figure 5 with stick spectra simulated for five low-lying isomers of HC_14_H_10_ using the B3LYP/6-311++G­(d,p) method. Difference spectra obtained after secondary irradiation at 315 and 223 nm are presented in Figures 5f and 5g, respectively, as these experiments yielded significant intensity variations. Bands in groups A, D, and E are marked with green, pink, and blue labels and lines, respectively. After irradiation at 315 nm, features in groups A and D increased by ∼30 and 8%, respectively, whereas those in group E decreased by ∼30% (Figure 5f). In contrast, following irradiation at 223 nm, group E increased by ∼26%, whereas groups A and D decreased by ∼29 and 11%, respectively (Figure 5g). A comparison of spectral features in groups A, D, and E across 575–1525 cm^–1^ with all seven HC_14_H_10_ isomers is provided in Figures S6–S8, respectively.

The observed wavenumbers of the bands in group A agree satisfactorily with predictions for 1-HC_14_H_10_ (Figure 5e and Figure S6d, in green) but align less well with other HC_14_H_10_ isomers (Figure 5 and Figure S6). The four most intense features, observed at 802.0, 654.4, 748.3, and 793.9 cm^–1^, align well with predicted vibrational wavenumbers (scaled harmonic) at 801, 654, 746, and 787 cm^–1^ (all of which are out-of-plane CH-bending modes). For the characteristic CH_2_-stretching (ν_10_ and ν_47_) modes, two bands observed at 2833.8 and 2814.6 cm^–1^ decreased upon irradiation at 223 nm and increased at 315 nm (Figure S3), consistent with scaled harmonic predictions at 2839 (symmetric CH_2_ stretch) and 2836 (antisymmetric CH_2_ stretch) cm^–1^, respectively. Additionally, six weaker bands also match the theoretical predictions. As summarized in Table 4 for modes with IR intensity >5 km mol^–1^, a total of 12 modes were identified for 1-HC_14_H_10_. All listed vibrational modes were observed, except for the CH-stretching region and two bands near 1507 (ν_15_) and 1415 (ν_19_) cm^–1^, which may be interfered by absorption of C_14_H_10_ at 1504.6 cm^–1^ and 4-HC_14_H_10_ (group B) at 1414.6 cm^–1^, respectively. Thus, these 12 features in group A are assigned to 1-HC_14_H_10_. The mean absolute deviation between observed and scaled harmonic vibrational wavenumbers for 1-HC_14_H_10_ is 5.8 ± 5.7 cm^–1^, with the largest deviations (22 and 25 cm^–1^) for ν_10_ and ν_47_, typical of the CH_2_-stretching modes in HPAH. The predicted stick spectrum based on anharmonic calculations of 1-HC_14_H_10_ (Figure S6e) shows poorer agreement than the scaled harmonic calculations, with a maximal deviation of 46 cm^–1^. No prominent combination or overtone bands were predicted for 1-HC_14_H_10_ except for two in the region 3050–3120 cm^–1^ (Table S2). The mean absolute deviation between the observed and anharmonic vibrational wavenumbers of 1-HC_14_H_10_ is 13.6 ± 15.0 cm^–1^.

**Table 4:** Comparison of Experimental Vibrational Wavenumbers (cm^–1^) and Relative IR Intensities (km mol^–1^) of 1- and 9-HC_14_H_10_ with Scaled Harmonic Vibrational Wavenumbers Predicted Using the B3YLP/6-311++G­(d,p) Method

1-HC_14_H_10_ (group A)						9-HC_14_H_10_ (group D)					
mode	sym.	calculation		*p*-H_2_		mode	sym.	calculation		*p*-H_2ν_	
ν_1_	a′	3098.9^a^	(14)^b^	^c^		ν_1_	a′	3088.5^a^	(20)^b^	^c^	
ν_2_	a′	3080.0	(5)	^c^		ν_2_	a′	3076.1	(11)	^c^	
ν_3_	a′	3067.4	(22)	^c^		ν_4_	a′	3064.9	(43)	^c^	
ν_4_	a′	3058.8	(32)	^c^		ν_5_	a′	3055.7	(14)	^c^	
ν_5_	a′	3057.5	(25)	^c^		ν_6_	a′	3051.5	(6)	^c^	
ν_6_	a′	3052.9	(6)	^c^		ν_7_	a′	3045.7	(17)	^c^	
ν_8_	a′	3039.2	(8)	^c^		ν_9_	a′	3035.1	(10)	^c^	
ν_9_	a′	3034.4	(7)	^c^		ν_10_	a′	2841.8	(38)	^d^	
ν_10_	a′	2839.2	(40)	2833.8	(25)^e^	ν_16_	a′	1472.4	(17)	1473.9	(8)^e^
ν_15_	a′	1507.4	(6)	^c^		ν_17_	a′	1456.4	(12)	1457.5	(2)
ν_19_	a′	1415.1	(9)	^f^		ν_18_	a′	1431.9	(7)	^c^	
ν_24_	a′	1275.8	(17)	1278.5	(13)	ν_20_	a′	1405.6	(6)	^g^	
ν_27_	a′	1197.2	(4)	1193.6	(2)	ν_26_	a′	1241.6	(8)	1243.2	(2)
ν_33_	a′	1053.0	(3)	1054.8	(2)	ν_35_	a′	1023.3	(6)	1027.8	(6)
ν_34_	a′	1034.7	(6)	1028.0	(2)	ν_36_	a′	989.7	(7)	993.4	(3)
ν_36_	a′	930.5	(10)	940.3	(2)	ν_41_	a′	619.9	(6)	^c^	
ν_47_	a″	2836.2	(15)	2814.6	(17)	ν_47_	a″	2839.5	(14)	^d^	
ν_54_	a″	855.5	(6)	862.1	(12)	ν_56_	a″	769.4	(8)	771.4	(6)
ν_55_	a″	801.0	(53)	802.0	(100)	ν_57_	a″	748.8	(98)	751.0	(100)
ν_56_	a″	786.7	(7)	793.9	(30)	ν_58_	a″	718.3	(39)	720.0	(35)
ν_57_	a″	745.9	(26)	748.3	(43)						
ν_59_	a″	653.7	(60)	654.4	(61)						

a Harmonic vibrational wavenumbers are scaled according to 0.9548*x* + 27.9 for wavenumbers >2000 cm^–1^ and 0.9804*x* + 2.3 for wavenumbers <2000 cm^–1^, in which *x* is the harmonic vibrational wavenumbers.

b Harmonic IR intensities (in km mol^–1^) are listed in parentheses. Only those with intensities >5 km mol^–1^ are listed.

c Obscured by C_14_H_10_.

d Obscured by group A.

e Percentage integrated intensities of each isomer are relative to the most intense band.

f Obscured by group B.

g Too weak to observe.

The relative IR intensities and vibrational wavenumbers observed for the bands in group D agree with those predicted for 9-HC_14_H_10_ (Figure 5h and Figure S7c, in pink) but do not match those of other HC_14_H_10_ isomers (Figure 5 and Figure S7). The two most intense features, observed at 751.0 and 720.2 cm^–1^, match the vibrational wavenumbers predicted at 749 and 718 cm^–1^ (both out-of-plane CH-bending modes). The characteristic CH_2_-stretching bands, predicted at 2840 (antisymmetric CH_2_-stretching mode) and 2842 cm^–1^ (symmetric CH_2_-stretching mode), were likely obscured by absorption bands of 1-HC_14_H_10_ (group A). Additionally, six weaker features align with the theoretical predictions. Table 4 lists all vibrational modes of 9-HC_14_H_10_ with an IR intensity greater than 5 km mol^–1^. Eight bands were assigned to 9-HC_14_H_10_. Except for the CH-stretching region, only three listed modes, predicted near 620, 1432, and 1406 cm^–1^, were not observed. These bands may have been obscured by absorption from C_14_H_10_ at 617.7 and 1432.1 cm^–1^ and from 4-HC_14_H_10_ (group B) at 1402.2 cm^–1^. Thus, the features in group D are assigned to 9-HC_14_H_10_. The average deviation between the experimental and predicted (scaled harmonic) vibrational wavenumbers for 9-HC_14_H_10_ is 2.3 ± 1.2 cm^–1^. The predicted stick spectrum based on anharmonic calculations of 9-HC_14_H_10_ is presented in Figure S7d. No prominent combination or overtone bands were predicted for 9-HC_14_H_10_, except in region 2850–3100 cm^–1^ (Table S2). The average deviation between the observed and anharmonic vibrational wavenumbers of 9-HC_14_H_10_ is 3.3 ± 2.6 cm^–1^.

The observed relative IR intensities and wavenumbers of the bands in group E agree with those predicted for 2-HC_14_H_10_ (Figure 5i and Figure S8e, in blue) but match less satisfactorily with those of other HC_14_H_10_ isomers (Figure 5 and Figure S8). The three features with the most significant intensities, observed at 745.6, 811.6, and 720.8 cm^–1^, match well with the predicted vibrational wavenumbers at 744, 809, and 722 cm^–1^ (all of which are out-of-plane CH-bending modes). Another medium-intensity out-of-plane CH-bending band, observed at 704.8 cm^–1^, aligns well with the prediction near 701 cm^–1^. The characteristic CH_2_-stretching bands, predicted at 2805 (antisymmetric) and 2819 cm^–1^ (symmetric), were observed at 2789.3 and 2818.9 cm^–1^, respectively. Furthermore, 12 weaker features also match the theoretical predictions. Table 5 summarizes all vibrational modes with an IR intensity >5 km mol^–1^. A total of 18 bands were observed for 2-HC_14_H_10_. All listed modes below 2850 cm^–1^ were observed, except one near 1375 cm^–1^ with an IR intensity of 5 km mol^–1^, which may have been too weak to detect. Thus, the features in group E are assigned to 2-HC_14_H_10_. The average deviation between experiments and predicted (scaled harmonic) vibrational wavenumbers for 2-HC_14_H_10_ is 4.4 ± 4.1 cm^–1^. The largest deviation between observed and predicted wavenumbers is 16 cm^–1^ for the ν_47_ (antisymmetric CH_2_-stretching) mode. The predicted stick spectrum based on anharmonic calculations of 2-HC_14_H_10_ is presented in Figure S8f; it agrees less satisfactorily with the scaled harmonic calculations, with a maximal deviation of 63 cm^–1^. No prominent combination or overtone bands were predicted for 2-HC_14_H_10_, except for one band at 2856 cm^–1^ with an IR intensity of 80 km mol^–1^ (Table S1). However, this band was not observed, likely indicating the limitation of simple anharmonic calculations. The average deviation between the experimental and anharmonic vibrational wavenumbers of 2-HC_14_H_10_ is 12.1 ± 17.4 cm^–1^.

### 4.4 Photolytic Behavior of Observed Hydrogenated Phenanthrene

After maintaining the matrix in darkness for 16 h, new (hydrogenated) species increased by 1–3 ppm, while protonated phenanthrene conformers decreased by 30–80 ppb, indicating that neutralization makes a minor contribution to the new species. However, this difference may be because only 80–130 ppb of the protonated species were produced after electron bombardment, whereas the initial mixing ratio of the parent was 32 ppm.

Although the primary purpose of secondary irradiation was to categorize absorption bands into distinct groups for spectral assignments, the photoresponses also provide hints regarding the photochemical behavior of individual isomers of HC_14_H_10_. Interpreting the photolytic response across the complete set of species and wavelengths remains difficult, mainly because computed IR intensities carry substantial uncertainties that undermine the accuracy of absolute mixing-ratio comparisons between different molecules. For this reason, only intraspecies trends are regarded as reliable. In addition, the extent of mixing-ratio reduction is tied to its initial magnitude; therefore, the stepwise percentage changes (shown in parentheses in Table 2) are taken as indicators of UV/vis absorption. These values, however, may be complicated by hydrogen migration from neighboring sites.

Absorption of a photon in the 423–223 nm range corresponds to an energy of 283–536 kJ mol^–1^, sufficient to dehydrogenate HC_14_H_10_ (requiring <97 kJ mol^–1^) or to induce hydrogen migration to adjacent carbon atoms, which requires <188 kJ mol^–1^ (Table 1 and Figure 2). It should be noted that we cannot distinguish hydrogen migration from hydrogen dissociation, followed by hydrogen addition to another site. In the following, when we state “hydrogen migration”, we refer to the results rather than the true mechanism. The vertical excitation spectra of 9-, 1-, 2-, 3-, and 4-HC_14_H_10_, predicted in the range of 470–200 nm, using the TD-B3LYP/6-311++G­(d,p) method, are illustrated in Figure S9. Time-dependent density functional theory is widely used for predicting electronic excitation energies, but its accuracy depends on the functional and nature of the excited state. Benchmark studies indicate that typical deviations from experimental transition energies are ∼0.2–0.5 eV for organic molecules.^29–31^ Accordingly, the calculated transition energies reported here should be interpreted primarily in terms of relative spectral trends rather than absolute wavelengths. The predicted oscillator strengths and wavelengths are summarized in Tables S6–S9. The calculated UV–vis spectra reveal how individual species respond to subsequent irradiation.

At 423 nm, only 2-HC_14_H_10_ (group E) decreased in mixing ratio by ∼0.9 ppm (−18%), while 1-HC_14_H_10_ (group A) and 4-HC_14_H_10_ (group B) increased by ∼0.5 and 0.2 ppm, respectively. 2-HC_14_H_10_ was predicted to exhibit a vertical absorption at 446 nm with an oscillator strength of 0.047. However, 1-HC_14_H_10_, 3-HC_14_H_10_, 4-HC_14_H_10_, and 9-HC_14_H_10_ were predicted to absorb at 414, 410, 435, and 408 nm, with oscillator strengths of 0.132, 0.042, 0.0037, and 0.072, respectively, which may extend over 423 nm. The increase in the level of 1-HC_14_H_10_ is likely due to hydrogen transfer from 2-HC_14_H_10_, while the increase in 4-HC_14_H_10_ may result from relay hydrogen transfer from site C2 to C4 via C3. During this process, 3-HC_14_H_10_ receives population from 2-HC_14_H_10_ and subsequently transfers a population comparable to that of 4-HC_14_H_10_.

Upon irradiation at 380 nm, the most prominent variations are a decrease in 3-C_14_H_10_ (group C) by ∼1.4 ppm (−29%) and an increase in 4-C_14_H_10_ (group B) by ∼1.2 ppm. This is consistent with a major absorption band of 3-HC_14_H_10_ near 384 nm (oscillator strength of 0.14) and hydrogen transfer from site C3 to site C4. A minor decrease of 1-C_14_H_10_ (group A) by ∼0.3 ppm (−8%) and a slight increase of 2-HC_14_H_10_ (group E) by ∼0.1 ppm are in line with absorption into two excited states of 1-HC_14_H_10_ at 357 and 384 nm (oscillator strengths 0.016 and 0.002).

After irradiation at 315 nm, the mixing ratios of 2-HC_14_H_10_ (group E) and 4-HC_14_H_10_ (group B) decreased by ∼1.3 and 0.8 ppm (−30 and −24%), respectively. In contrast, those of 1-HC_14_H_10_ (group A) and 9-HC_14_H_10_ (group D) increased by ∼0.9 and 0.3 ppm, respectively. 2-HC_14_H_10_ was predicted to absorb near 333 and 314 nm with oscillator strengths of 0.075 and 0.028, respectively, whereas 4-HC_14_H_10_ was predicted to absorb near 352 and 329 nm with oscillator strengths of 0.156 and 0.006, respectively. The hydrogen transfer from 2-HC_14_H_10_ to 1-HC_14_H_10_ is plausible, but the decrease in 4-HC_14_H_10_ remains unexplained. The slight increase in 9-HC_14_H_10_ might be attributed to a relay hydrogen transfer from position C2 via C1 to C10 (equivalent to C9).

At 223 nm, the mixing ratios of 1-HC_14_H_10_ (group A) and 9-HC_14_H_10_ (group D) decreased by ∼1.1 and 0.5 ppm (−29 and −11%), respectively, whereas that of 2-HC_14_H_10_ (group E) increased by ∼0.8 ppm. The decrease in 1-HC_14_H_10_ may be attributed to intense bands predicted near 218, 219, 221, and 223 nm with oscillator strengths of 0.230, 0.165, 0.397, and 0.077, respectively. In contrast, 2-HC_14_H_10_ has no intense absorption in this spectral region. The decrease in the level of 9-HC_14_H_10_ might be attributed to an intense band predicted near 224 nm with an oscillator strength of 0.362. The hydrogen transfer from 1-HC_14_H_10_ to 2-HC_14_H_10_ is conceivable, but that from 9-HC_14_H_10_ is not obvious. One possibility is a relay hydrogen transfer from position C10 (equivalent to C9) via the C1 to C2. The smaller decreases of 3-HC_14_H_10_ and 4-HC_14_H_10_ by ∼0.2 and 0.1 ppm (−5 and −4%) may be rationalized by the predicted vertical transitions of 3-HC_14_H_10_ and 4-HC_14_H_10_ at 230/231 and 214 nm, with oscillator strengths of 0.578/0.145 and 0.487, respectively.

**Table 5:** Comparison of Experimental Vibrational Wavenumbers (cm^–1^) and Relative IR Intensities (km mol^–1^) of 2-HC_14_H_10_ with Scaled Harmonic Vibrational Wavenumbers Predicted with the B3YLP/6-311++G­(d,p) Method

2-HC_14_H_10_ (group E)					
mode	sym.	calculation		*p*-H_2_	
ν_1_	a′	3087.7^a^	(22)^b^	^c^	
ν_2_	a′	3071.7	(17)	^c^	
ν_3_	a′	3067.1	(11)	^c^	
ν_4_	a′	3057.9	(32)	^c^	
ν_5_	a′	3051.8	(11)	^c^	
ν_6_	a′	3043.3	(8)	^c^	
ν_7_	a′	3042.2	(5)	^c^	
ν_8_	a′	3041.5	(14)	^c^	
ν_10_	a′	2819.0	(70)	2818.9	(39)^d^
ν_15_	a′	1514.4	(7)	1515.2	(16)
ν_17_	a′	1431.7	(2)	1434.4	(11)
ν_20_	a′	1407.5	(9)	1397.7	(20)
ν_21_	a′	1374.5	(5)	^e^	
ν_23_	a′	1331.3	(7)	1329.1	(8)
ν_26_	a′	1241.3	(7)	1242.2	(7)
ν_31_	a′	1145.3	(2)	1140.0	(10)
ν_33_	a′	1039.5	(9)	1038.2	(10)
ν_36_	a′	897.0	(15)	905.0	(22)
ν_41_	a′	591.7	(7)	588.9	(14)
ν_47_	a″	2804.8	(17)	2789.3	(19)
ν_53_	a″	912.7	(10)	907.5	(14)
ν_54_	a″	851.0	(7)	856.6	(21)
ν_55_	a″	809.2	(39)	811.6	(75)
ν_56_	a″	778.6	(3)	788.0	(10)
ν_57_	a″	743.9	(36)	745.6	(100)
ν_58_	a″	721.9	(34)	720.8	(73)
ν_59_	a″	700.8	(18)	704.8	(29)

a Harmonic vibrational wavenumbers are scaled according to 0.9548*x* + 27.86 for wavenumbers >2000 cm^–1^ and 0.9804*x* + 2.33 for wavenumbers <2000 cm^–1^, in which *x* is the harmonic vibrational wavenumbers.

b Harmonic IR intensities (in km mol^–1^) are listed in parentheses. Only those with intensities >5 km mol^–1^ are listed.

c Obscured by C_14_H_10_.

d Percentage integrated intensities of each isomer are relative to the most intense band.

e Too weak to observe.

### 4.5 Implications to UIR Identification

Figure 6 contrasts the stick IR spectra measured for 9-, 1-, 2-, 3-, and 4-HC_14_H_10_ with the unidentified infrared (UIR) features detected in the Orion Bar PDR.^32,33^ In astrophysical environments, however, PAH emission arises from highly vibrationally excited molecules that relax via an infrared cascade after absorbing energetic UV photons. Because of the anharmonic nature of the vibrational potential, transitions from higher vibrational levels occur at slightly lower frequencies than the fundamental transition, leading to a progressive redshift of emission from highly vibrationally excited states. The cumulative effect of these hot-band transitions induces asymmetric band profiles with extended red wings in astronomical spectra.^34^ To generate simulated spectra, the experimental stick data for each compound were broadened using a Lorentzian profile with a full width at half-maximum of 20 cm^–1^. Furthermore, to compensate for missing contributions obscured by spectral overlap, additional convoluted spectra derived from scaled harmonic frequencies and harmonic intensities are displayed as lighter curves. Taken together, the calculated spectra of hydrogenated phenanthrene reproduce the experimental results with satisfactory fidelity, except for a limited number of missing bands that could not be identified due to overlap.

**Figure 6:**
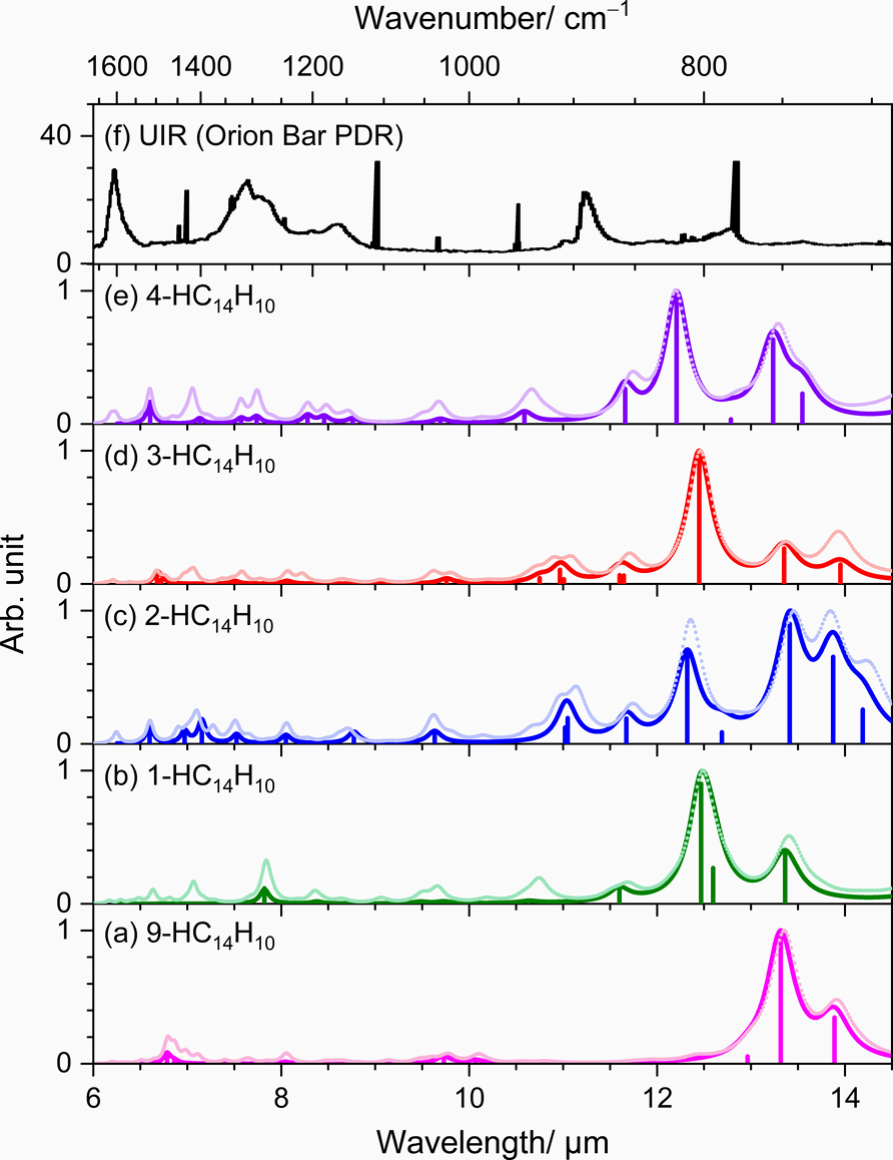
Comparison of UIR emission bands with IR spectra of HC_14_H_10_. IR spectra of (a) 9-HC_14_H_10_, (b) 1-HC_14_H_10_, (c) 2-HC_14_H_10_, (d) 3-HC_14_H_10_, and (e) 4-HC_14_H_10_. Observed stick spectra are convoluted with a Lorentzian function (FWHM = 20 cm^–1^) to produce spectra of the same color. For each species, spectra with a light color are convoluted from scaled harmonic vibrational stick spectra with a Lorentzian function (FWHM = 20 cm^–1^). (e) UIR emission spectrum from Orion Bar PDR. Reproduced from Peeters et al.^33^ Copyright [2021] American Chemical Society.

The five monohydrogenated phenanthrene isomers (Figure 6a–e) exhibit pronounced absorption features in the 12–14 μm region, dominated by out-of-plane CH-bending vibrations, along with weaker signals between 6 and 12 μm arising from CC-stretching, out-of-plane CH-bending, and in-plane CH-bending modes. These spectral characteristics, however, show poor correspondence with the UIR bands displayed in Figure 6f, indicating that hydrogenated phenanthrene is not a likely contributor to the UIR signatures. This inference is consistent with the fact that HC_14_H_10_ is insufficiently large to survive the harsh UV radiation prevalent in interstellar environments. In addition, hydrogenated PAHs possess open-shell electronic configurations, rendering them relatively unstable and prone to fragmentation or further reactions with other cosmic species.

## 5 Conclusions

Electron bombardment during the deposition of phenanthrene (C_14_H_10_) and *p*-H_2_ onto a cold substrate yielded several products. In addition to the previously reported protonated phenanthrene isomers,^15^ five isomers of monohydrogenated phenanthrene (9-, 1-, 2-, 3-, and 4-HC_14_H_10_) were formed. The IR bands of these hydrogenated species intensified after prolonged dark storage of the matrix, as the protonated phenanthrene was neutralized by trapped electrons, and H atoms in the matrix diffused to react with C_14_H_10_. These features were classified into five distinct groups based on their photochemical responses to irradiation at 423, 380, 315, and 223 nm. Assignments of these features to 9-, 1-, 2-, 3-, and 4-HC_14_H_10_ were supported by comparison with harmonic vibrational wavenumbers (scaled) and IR intensities calculated using the B3LYP/6-311++G­(d,p) method. This study presents the first report of the IR spectra for these five monohydrogenated phenanthrene isomers. Furthermore, their photolytic behavior at various wavelengths was rationalized using electronic vertical excitation spectra predicted by quantum-chemical computations together with a mechanism of hydrogen migration to neighboring sites. Consistent with the expectation that HC_14_H_10_ is too small to survive intense UV radiation, the observed IR spectra of 9-, 1-, 2-, 3-, and 4-HC_14_H_10_ indicate that these species are unlikely to be significant carriers of the UIR bands.

## Supplementary Material



## Notes

Cartesian coordinates of 9-, 1-, 2-, 3-, 4-, 4a-, and 8a-HC_14_H_10_; scaled harmonic vibrational wavenumbers and IR intensities of 9-, 1-, 2-, 3-, 4-, 4a-, and 8a-HC_14_H_10_; estimated mixing ratio of C_14_H_10_ and 9-, 1-, 2-, 3-, and 4-HC_14_H_10_; predicted vertical excitation wavelengths and oscillator strengths of electronic excitation of C_14_H_10_ and 9-, 1-, 2-, 3-, 4-, 4a-, and 8a-HC_14_H_10_ (nine tables); geometries and relative energies of seven possible isomers of HC_14_H_10_; infrared spectra in the range 575–1525 cm^–1^ of an electron-bombarded C_14_H_10_/*p*-H_2_ matrix after each experimental step; comparison of observed IR spectra in region 2640–3120 cm^–1^ with stick spectra of seven isomers of HC_14_H_10_; comparison of bands in groups A, B, C, D, and E in region 575–1525 cm^–1^ with stick spectra of seven isomers of HC_14_H_10_; and calculated vertical electronic excitation spectra of 9-, 1-, 2-, 3-, and 4-HC_14_H_10_ (nine figures) (PDF)
