# Supplementary material for: Infrared Characterization of Mono-Hydrogenated Phenanthrene Isomers (1‑, 2‑, 3‑, 4‑, and 9‑HC14H10) in Solid para-Hydrogen
Source: J Phys Chem A. 2026 Apr 2;130(15):2957–67. doi: 10.1021/acs.jpca.5c08413 (PMC13093475; doi:10.1021/acs.jpca.5c08413)
Supplement: Supplementary file 1 [file jp5c08413_si_001.pdf]

Supporting Information

Infrared Characterization of Mono-Hydrogenated Phenanthrene Isomers (1-, 2-, 3-, 4-, and 9-$HC_{14}H_{10}$) in Solid *Para*-Hydrogen

Jun-Ying Feng[†] and Yuan-Pern Lee[†,‡*]

[†]*Department of Applied Chemistry and Institute of Molecular Science, National Yang Ming Chiao Tung University, Hsinchu 300093, Taiwan*

[‡]*Center for Emergent Functional Matter Science, National Yang Ming Chiao Tung University, Hsinchu 300093, Taiwan*

**E-mail: yplee@nycu.edu.tw* (Y.-P. Lee)

Table of Contents

Table S1. Cartesian Coordinates of Optimized Geometries of Seven Conformers of $HC_{14}H_{10}$..............S1

Table S2. Scaled Harmonic and Anharmonic Vibrational Wavenumbers and IR Intensities of 9-, 1-, and 2-$HC_{14}H_{10}$ Predicted Using the B3LYP/6-311++G(d,p) Method S4

Table S3. Scaled Harmonic and Anharmonic Vibrational Wavenumbers and IR Intensities of 3- and 4-$HC_{14}H_{10}$ Predicted Using the B3LYP/6-311++G(d,p) Method..S8

Table S4. Scaled Harmonic Vibrational Wavenumbers and IR Intensities of 4a- and 8a-$HC_{14}H_{10}$ Predicted Using the B3LYP/6-311++G(d,p) Method...S11

Table S5. Estimated Mixing Ratios of Each Species in the Electron-bombarded $C_{14}H_{10}$/*p*-H_2 Matrix Experiment and Integration Ranges S14

Table S6. Vertical Excitation Wavelengths and Oscillator Strengths of Electronic Excitations of $C_{14}H_{10}$ and 9-$HC_{14}H_{10}$ Predicted Using the TD-B3LYP/6-311++G(d,p) Method...S15

Table S7. Vertical Excitation Wavelengths and Oscillator Strengths of Electronic Excitations of 1- and 2-$HC_{14}H_{10}$ Predicted Using the TD-B3LYP/6-311++G(d,p) Method..S17

Table S8. Vertical Excitation Wavelengths and Oscillator Strengths of Electronic Excitations of 3- and 4-$HC_{14}H_{10}$ Predicted Using the TD-B3LYP/6-311++G(d,p) Method..S19

Table S9. Vertical Excitation Wavelengths and Oscillator Strengths of Electronic Excitations of 4a- and 8a-$HC_{14}H_{10}$ Predicted Using the TD-B3LYP/6-311++G(d,p) Method..S21

Figure S1. Geometries and relative energies of seven isomers of $HC_{14}H_{10}$. S23

Figure S2. Infrared spectra (in the region 575–1525 cm^{-1}) of an electron-bombarded $C_{14}H_{10}$/*p*-H_2 matrix after each experimental step. S24

Figure S3. Comparison of observed IR spectra in the region 2640–3120 cm^{-1} with stick spectra of seven isomers of $HC_{14}H_{10}$...S26

Figure S4. Comparison of bands in group B in the region 575−1525 cm^{-1} with stick spectra of seven isomers of $HC_{14}H_{10}$...S27

Figure S5. Comparison of bands in group C in the region 575−1525 cm^{-1} with stick spectra of seven isomers of $HC_{14}H_{10}$...S29

Figure S6. Comparison of bands in group A in the region 575−1525 cm^{-1} with stick spectra of seven isomers of $HC_{14}H_{10}$...S31

Figure S7. Comparison of bands in group D in the region 575−1525 cm^{-1} with stick spectra of seven isomers of $HC_{14}H_{10}$...S33

Figure S8. Comparison of bands in group E in the region 575−1525 cm^{-1} with stick spectra of seven isomers of $HC_{14}H_{10}$..S35
Figure S9. Calculated vertical electronic excitation spectra of 9-, 1-, 2-, 3-, and 4-$HC_{14}H_1$ S37

Table S1. Cartesian Coordinates of Optimized Geometries of Seven Conformers of $HC_{14}H_{10}$

	X	Y	Z	X	Y	Z
		9-$HC_{14}H_{10}$			1-$HC_{14}H_{10}$	
C	3.580256	-0.336210	-0.000200	3.600040	-0.294410	0.000001
C	2.890272	-1.558860	0.000260	2.916228	-1.527130	0.000000
C	2.873533	0.846228	-0.000420	2.884049	0.879262	0.000000
C	1.497292	-1.572850	0.000418	1.538312	-1.562550	0.000000
C	1.446401	0.860944	-0.000180	1.465996	0.872125	0.000000
C	0.750896	2.072941	-0.000230	0.766501	-0.374280	0.000000
C	0.743573	-0.395280	0.000153	0.727336	2.085949	-0.000001
C	-0.733000	2.161289	0.000481	-0.645520	2.066655	-0.000001
C	-0.734150	-0.392050	0.000086	-0.685480	-0.376070	0.000000
C	-1.444490	0.827032	0.000161	-1.367460	0.856073	0.000000
C	-1.480280	-1.587790	-0.000160	-1.443550	-1.586670	0.000000
C	-2.843000	0.816488	0.000017	-2.854590	-1.573390	0.000000
C	-2.866670	-1.583880	-0.000280	-2.880750	0.919822	0.000001
C	-3.559960	-0.372410	-0.000190	-3.565490	-0.409100	0.000001
H	4.664501	-0.323280	-0.000360	4.684020	-0.275510	0.000001
H	3.437506	-2.494290	0.000482	3.477778	-2.454680	0.000000
H	3.396676	1.796795	-0.000740	3.397182	1.835453	0.000001
H	1.314109	3.000568	-0.000550	1.264907	3.028086	-0.000001
H	0.999464	-2.533850	0.000765	1.046233	-2.526060	-0.000001
H	-0.970460	-2.542140	-0.000300	-0.939830	-2.542650	-0.000001
H	-1.069810	2.749652	0.869513	-1.195820	3.002939	-0.000001
H	-1.070660	2.750597	-0.867550	-3.218290	1.509931	0.867879
H	-3.373940	1.763925	0.000068	-3.218290	1.509932	-0.867880
H	-3.407500	-2.523590	-0.000470	-3.381750	-2.522130	-0.000001
H	-4.643920	-0.357990	-0.000310	-4.649910	-0.423090	0.000000
		2-$HC_{14}H_{10}$			3-$HC_{14}H_{10}$	
C	3.616221	-0.287000	0.000000	3.566275	-0.158770	0.000000
C	2.819504	-1.554540	0.000000	2.955891	-1.525560	0.000000
C	2.762021	0.936485	0.000000	2.809779	0.953172	0.000000
C	1.471814	-1.565650	0.000000	1.459835	-1.517000	0.000000
C	1.374386	0.895674	0.000000	1.363673	0.914167	0.000000
C	0.674444	-0.357760	0.000000	0.697327	-0.363250	0.000000
C	0.607818	2.116683	0.000000	0.615794	2.105566	0.000000
C	-0.748210	2.099320	0.000000	-0.759550	2.094685	0.000000
C	-0.760400	-0.375470	0.000000	-0.771070	-0.376390	0.000000
C	-1.478790	0.865057	0.000000	-1.480530	0.861248	0.000000
C	-1.524770	-1.573290	0.000000	-1.523640	-1.568620	0.000000

C	-2.887710	0.858558	0.000000	-2.895560	0.851449	0.000000
C	-2.904030	-1.549250	0.000000	-2.906500	-1.553780	0.000000
C	-3.598750	-0.325790	0.000000	-3.601590	-0.332430	0.000000
H	4.301708	-0.275210	0.866163	4.649022	-0.086340	0.000000
H	4.301707	-0.275210	-0.866160	3.327540	-2.101420	-0.865990
H	3.366110	-2.492160	0.000000	3.327540	-2.101420	0.865994
H	3.257747	1.902117	0.000000	3.283219	1.930648	0.000000
H	1.146058	3.058816	0.000000	1.148270	3.051513	0.000000
H	0.968776	-2.524410	0.000000	0.979877	-2.487590	0.000000
H	-1.024730	-2.532720	0.000000	-1.018410	-2.525930	0.000000
H	-1.308650	3.028663	0.000000	-1.317140	3.024716	0.000000
H	-3.411290	1.809244	0.000000	-3.420310	1.801291	0.000000
H	-3.456230	-2.482520	0.000000	-3.454710	-2.489160	0.000000
H	-4.682590	-0.314780	0.000000	-4.685720	-0.323270	0.000000
		$4\text{-}HC_{14}H_{10}$			$4a\text{-}HC_{14}H_{10}$	
C	3.564938	-0.227180	0.000000	3.549118	-0.301080	-0.232680
C	2.967146	-1.455830	0.000000	2.884355	-1.522380	-0.130430
C	2.800819	0.954298	0.000000	2.826229	0.880241	-0.143770
C	1.478813	-1.617140	0.000000	1.502546	-1.551290	0.067711
C	1.372066	0.909089	0.000000	1.433026	0.866591	0.051701
C	0.695008	-0.321740	0.000000	0.760833	-0.375870	0.164015
C	0.617443	2.125321	0.000000	0.680623	2.101912	0.082560
C	-0.731820	-0.348700	0.000000	-0.687140	2.110811	0.046371
C	-0.745390	2.114207	0.000000	-0.731930	-0.365810	0.500414
C	-1.463930	0.883926	0.000000	-1.441310	0.914024	0.087047
C	-1.476270	-1.561400	0.000000	-1.510550	-1.604710	0.139930
C	-2.853580	-1.555790	0.000000	-2.816300	0.888214	-0.171760
C	-2.877630	0.855249	0.000000	-2.827320	-1.549780	-0.172110
C	-3.565560	-0.338300	0.000000	-3.519490	-0.300960	-0.274460
H	4.647798	-0.156600	0.000000	4.622301	-0.273050	-0.383790
H	3.567460	-2.359250	0.000000	3.436099	-2.452780	-0.202430
H	3.292193	1.920342	0.000000	3.332652	1.835953	-0.232650
H	1.181784	-2.229360	-0.867650	1.231870	3.034799	0.033031
H	1.181783	-2.229360	0.867652	1.014387	-2.513970	0.151468
H	1.157324	3.066529	0.000000	-0.743610	-0.328690	1.616139
H	-0.955320	-2.510370	0.000000	-1.017680	-2.565400	0.213519
H	-1.303680	3.044667	0.000000	-1.217730	3.051748	-0.065440
H	-3.395740	-2.494950	0.000000	-3.325370	1.831466	-0.346970
H	-3.416270	1.797421	0.000000	-3.370120	-2.468260	-0.369830
H	-4.649570	-0.345150	0.000000	-4.578950	-0.291280	-0.500320
		$8a\text{-}HC_{14}H_{10}$				

C	3.580879	-0.240900	-0.064580
C	2.950750	-1.475610	0.119395
C	2.810632	0.910705	-0.171980
C	1.566944	-1.549770	0.188182
C	1.413129	0.858922	-0.093630
C	0.764002	-0.396450	0.083832
C	0.611645	2.069154	-0.238800
C	-0.690090	-0.440190	0.112231
C	-0.704690	2.078720	0.001228
C	-1.422360	0.837673	0.480735
C	-1.428250	-1.582500	-0.162140
C	-2.822340	-1.567250	-0.256750
C	-2.888490	0.817171	0.142864
C	-3.535410	-0.337360	-0.153170
H	4.661754	-0.181320	-0.119920
H	3.542940	-2.378600	0.217234
H	3.291755	1.872314	-0.319520
H	1.121147	2.977970	-0.544080
H	1.099427	-2.513440	0.350551
H	-0.909870	-2.510570	-0.376010
H	-1.285000	2.991142	-0.092380
H	-1.375410	0.897266	1.596446
H	-3.355540	-2.479810	-0.494210
H	-3.426160	1.758046	0.202778
H	-4.603190	-0.326860	-0.345390

Table S2. Scaled Harmonic and Anharmonic Vibrational Wavenumbers and IR Intensities of 9-, 1-, and 2-$HC_{14}H_{10}$ Predicted Using the B3LYP/6-311++G(d,p) Method

mode	sym.	9-$HC_{14}H_{10}$				1-$HC_{14}H_{10}$				2-$HC_{14}H_{10}$			
		calculation[a]				calculation[a]				calculation[a]			
		harmonic		anharmonic		harmonic		anharmonic		harmonic		anharmonic	
ν_1	a′	3088.5	(20.3)	3079.9	(41.5)	3098.9	(14.4)	3103.7	(10.5)	3087.7	(21.6)	3093.3	(3.7)
ν_2	a′	3076.1	(11.1)	3075.7	(5.7)	3080.0	(4.6)	3058.5	(9.3)	3071.7	(16.5)	3068.3	(6.3)
ν_3	a′	3068.5	(0.8)	3065.2	(45.9)	3067.4	(22.4)	3074.3	(12.4)	3067.1	(11.3)	3048.5	(6.4)
ν_4	a′	3064.9	(42.6)	3061.0	(58.8)	3058.8	(31.8)	3043.0	(31.6)	3057.9	(31.5)	3032.5	(11.2)
ν_5	a′	3055.7	(13.9)	3033.6	(100.4)	3057.5	(25.1)	3047.5	(2.5)	3051.8	(11.1)	3075.6	(57.6)
ν_6	a′	3051.5	(6.0)	3038.1	(64.6)	3052.9	(5.9)	3033.6	(1.1)	3043.3	(8.1)	3018.8	(3.2)
ν_7	a′	3045.7	(17.1)	3056.3	(0.5)	3042.8	(0.7)	3014.1	(17.6)	3042.2	(4.9)	3004.4	(15.0)
ν_8	a′	3041.5	(1.7)	3005.5	(10.1)	3039.2	(7.5)	3031.2	(37.2)	3041.5	(13.5)	3026.7	(6.4)
ν_9	a′	3035.1	(9.6)	3011.9	(9.1)	3034.4	(7.2)	3003.8	(8.5)	3038.3	(0.9)	3030.6	(5.8)
ν_{10}	a′	2841.8	(38.2)	2792.0	(18.6)	2839.2	(39.7)	2787.4	(16.3)	2819.0	(69.6)	2755.5	(9.5)
ν_{11}	a′	1611.8	(1.4)	1602.5	(1.5)	1621.2	(1.2)	1610.8	(0.6)	1619.4	(0.2)	1607.8	(0.8)
ν_{12}	a′	1582.4	(0.5)	1574.4	(0.1)	1589.7	(1.3)	1582.2	(0.2)	1601.6	(2.6)	1590.1	(3.1)
ν_{13}	a′	1574.1	(0.4)	1564.3	(0.2)	1561.4	(0.7)	1548.9	(0.1)	1599.7	(1.2)	1593.5	(2.1)
ν_{14}	a′	1536.1	(2.0)	1526.1	(1.5)	1542.4	(1.8)	1532.1	(0.7)	1537.3	(0.5)	1527.2	(0.6)
ν_{15}	a′	1505.1	(1.7)	1496.2	(1.6)	1507.4	(5.7)	1497.7	(4.0)	1514.4	(7.4)	1507.3	(4.3)
ν_{16}	a′	1472.4	(16.9)	1467.8	(7.3)	1468.6	(1.8)	1464.7	(1.6)	1449.1	(4.0)	1444.4	(2.0)
ν_{17}	a′	1456.4	(11.6)	1453.5	(11.1)	1431.4	(1.0)	1426.1	(0.3)	1431.7	(2.2)	1431.8	(1.5)
ν_{18}	a′	1431.9	(7.2)	1435.4	(1.4)	1421.2	(0.1)	1425.6	(2.0)	1421.0	(2.4)	1412.2	(0.9)
ν_{19}	a′	1421.2	(0.4)	1407.9	(0.2)	1415.1	(8.7)	1395.0	(5.0)	1415.3	(1.0)	1417.1	(0.6)
ν_{20}	a′	1405.6	(5.5)	1398.0	(1.2)	1395.6	(1.1)	1391.5	(0.5)	1407.5	(8.6)	1387.3	(7.6)
ν_{21}	a′	1351.1	(2.2)	1340.6	(0.9)	1372.1	(0.9)	1367.6	(0.7)	1374.5	(4.7)	1362.3	(1.5)
ν_{22}	a′	1314.8	(1.6)	1301.0	(0.3)	1353.2	(0.1)	1346.3	(0.2)	1342.1	(1.0)	1336.0	(1.4)

ν_{23}	a′	1305.7	(2.7)	1300.4	(1.4)	1304.8	(1.2)	1304.1	(0.5)	1331.3	(6.5)	1325.2	(2.8)
ν_{24}	a′	1278.7	(0.3)	1278.1	(0.3)	1275.8	(17.3)	1268.4	(7.1)	1305.9	(1.9)	1306.0	(0.5)
ν_{25}	a′	1273.5	(1.1)	1273.8	(0.2)	1266.3	(2.0)	1268.7	(5.1)	1272.2	(0.5)	1273.8	(0.3)
ν_{26}	a′	1241.6	(7.6)	1242.7	(5.1)	1224.6	(0.7)	1223.2	(0.1)	1241.3	(6.5)	1241.8	(4.5)
ν_{27}	a′	1195.2	(0.0)	1196.2	(0.0)	1197.2	(4.1)	1197.1	(2.6)	1207.6	(1.1)	1208.5	(1.6)
ν_{28}	a′	1181.6	(1.5)	1177.3	(0.7)	1190.9	(1.1)	1192.1	(0.2)	1178.4	(0.7)	1181.6	(0.3)
ν_{29}	a′	1166.7	(1.1)	1173.6	(1.1)	1161.8	(0.3)	1167.6	(0.2)	1160.9	(1.4)	1165.6	(1.0)
ν_{30}	a′	1154.3	(1.5)	1160.2	(1.7)	1157.9	(1.0)	1161.0	(0.6)	1149.4	(2.0)	1153.3	(1.9)
ν_{31}	a′	1133.4	(0.7)	1137.1	(0.2)	1149.9	(0.4)	1156.1	(0.1)	1145.3	(2.3)	1150.1	(2.3)
ν_{32}	a′	1093.9	(2.1)	1093.5	(0.9)	1103.4	(2.1)	1104.4	(1.4)	1094.4	(0.3)	1098.1	(0.1)
ν_{33}	a′	1055.6	(2.4)	1057.7	(2.4)	1053.0	(3.4)	1056.3	(1.2)	1039.5	(8.9)	1039.3	(0.2)
ν_{34}	a′	1041.8	(2.6)	1044.6	(1.4)	1034.7	(6.4)	1038.9	(3.6)	1018.5	(2.0)	1018.7	(0.9)
ν_{35}	a′	1023.3	(6.4)	1027.2	(5.1)	981.7	(1.6)	983.1	(0.4)	985.6	(1.6)	988.4	(1.4)
ν_{36}	a′	989.7	(6.6)	993.8	(5.9)	930.5	(9.6)	937.4	(2.9)	897.0	(14.7)	898.4	(13.0)
ν_{37}	a′	848.6	(0.2)	849.5	(0.1)	867.7	(2.6)	871.7	(1.3)	855.2	(2.8)	858.4	(2.2)
ν_{38}	a′	804.9	(3.1)	807.4	(1.9)	821.3	(2.3)	825.6	(2.3)	810.3	(0.4)	814.3	(0.4)
ν_{39}	a′	701.3	(0.2)	704.6	(0.4)	704.9	(0.9)	708.2	(0.8)	704.5	(0.5)	708.0	(0.3)
ν_{40}	a′	685.8	(0.8)	686.7	(0.5)	686.6	(0.4)	689.1	(0.2)	689.7	(0.4)	692.5	(0.4)
ν_{41}	a′	619.9	(5.6)	623.8	(5.4)	597.1	(2.8)	602.9	(0.3)	591.7	(7.1)	594.4	(5.7)
ν_{42}	a′	540.3	(0.1)	547.7	(0.1)	535.6	(1.1)	536.5	(0.6)	537.8	(0.7)	540.5	(0.6)
ν_{43}	a′	487.8	(0.1)	494.1	(0.1)	483.8	(0.1)	487.4	(0.1)	485.3	(0.4)	489.0	(0.5)
ν_{44}	a′	425.9	(0.2)	427.0	(0.3)	435.7	(2.1)	438.1	(1.9)	437.0	(1.9)	438.8	(1.7)
ν_{45}	a′	391.2	(0.0)	392.1	(0.0)	392.4	(0.5)	394.2	(0.5)	400.5	(0.6)	402.9	(0.7)
ν_{46}	a′	245.4	(0.3)	244.7	(0.4)	240.6	(0.8)	239.7	(0.7)	240.4	(0.7)	238.0	(0.5)
ν_{47}	a″	2839.5	(14.0)	2770.3	(43.7)	2836.2	(15.0)	2774.3	(20.6)	2804.8	(17.2)	2737.6	(19.8)
ν_{48}	a″	1177.9	(0.1)	1170.9	(0.0)	1181.1	(0.4)	1173.9	(0.1)	1160.3	(0.4)	1149.9	(0.3)
ν_{49}	a″	970.4	(0.0)	1000.5	(0.0)	971.3	(0.1)	1004.9	(0.0)	968.5	(0.0)	971.9	(0.0)
ν_{50}	a″	957.1	(0.0)	984.1	(0.0)	952.8	(0.0)	954.5	(0.3)	964.4	(0.2)	967.8	(0.0)

ν_{51}	a″	945.8	(1.0)	960.2	(0.2)	948.1	(0.4)	955.9	(2.4)	958.3	(0.0)	996.5	(1.1)
ν_{52}	a″	925.4	(1.3)	942.4	(1.0)	943.9	(1.6)	966.2	(0.3)	937.3	(3.0)	950.8	(0.3)
ν_{53}	a″	911.9	(0.2)	909.1	(0.3)	917.5	(0.2)	910.6	(0.0)	912.7	(10.4)	907.4	(14.9)
ν_{54}	a″	850.6	(0.5)	855.4	(0.1)	855.5	(5.6)	881.2	(2.5)	851.0	(7.4)	878.2	(1.2)
ν_{55}	a″	837.4	(1.1)	850.8	(0.7)	801.0	(53.4)	805.1	(40.4)	809.2	(38.9)	821.4	(28.8)
ν_{56}	a″	769.4	(7.5)	764.5	(7.1)	786.7	(6.6)	802.2	(3.2)	778.6	(2.5)	793.3	(12.1)
ν_{57}	a″	748.8	(97.5)	755.8	(58.3)	745.9	(25.8)	763.1	(34.6)	743.9	(36.4)	748.4	(29.4)
ν_{58}	a″	718.3	(38.5)	717.1	(43.4)	728.6	(1.7)	728.0	(4.6)	721.9	(34.2)	715.4	(36.0)
ν_{59}	a″	705.8	(2.4)	705.1	(20.8)	653.7	(59.8)	656.2	(50.3)	700.8	(18.1)	695.8	(22.7)
ν_{60}	a″	663.0	(1.3)	658.2	(0.4)	645.8	(2.8)	648.8	(0.0)	648.6	(2.4)	635.6	(1.3)
ν_{61}	a″	554.7	(0.2)	541.0	(0.3)	538.5	(1.1)	540.2	(1.6)	534.8	(0.0)	531.7	(0.1)
ν_{62}	a″	491.1	(4.0)	482.7	(3.5)	500.2	(0.8)	490.7	(0.3)	513.5	(0.3)	506.1	(0.5)
ν_{63}	a″	442.3	(0.0)	437.7	(0.0)	467.0	(2.3)	464.2	(2.1)	434.0	(14.5)	429.5	(14.0)
ν_{64}	a″	421.2	(7.5)	415.9	(7.7)	405.5	(3.0)	402.0	(3.8)	384.2	(0.4)	379.5	(0.5)
ν_{65}	a″	273.8	(0.1)	269.3	(0.2)	284.7	(5.2)	278.6	(5.4)	292.9	(0.3)	285.7	(0.7)
ν_{66}	a″	219.3	(1.7)	217.0	(2.0)	224.2	(2.1)	222.5	(1.8)	216.1	(4.8)	213.7	(3.9)
ν_{67}	a″	170.7	(5.1)	175.6	(4.8)	131.6	(0.2)	144.5	(0.0)	173.4	(0.0)	169.5	(0.0)
ν_{68}	a″	89.9	(0.2)	91.3	(0.3)	96.8	(1.0)	96.8	(1.2)	98.1	(0.5)	97.0	(0.4)
ν_{69}	a″	35.3	(0.3)	57.0	(0.0)	70.4	(0.2)	68.3	(0.1)	74.7	(0.8)	75.4	(0.4)
			overtone/combination				overtone/combination				overtone/combination		
			$2\nu_{12}$	3152.4	(5.7)		$2\nu_{19}$	2801.4	(7.9)		$2\nu_{20}$	2793.2	(5.3)
			$2\nu_{19}$	2823.7	(6.2)		$2\nu_{49}$	2017.8	(6.2)		$\nu_{14} +$	3127.4	(11.6)
			$2\nu_{49}$	2012.8	(6.8)		$\nu_{14} +$	3116.8	(7.1)		$\nu_{15} +$	3103.9	(4.4)
			$2\nu_{50}$	1976.7	(7.0)		$\nu_{14} +$	3084.7	(8.9)		$\nu_{15} +$	3101.0	(17.6)
			$\nu_{15} + \nu_{1}$	3097.7	(6.5)		$\nu_{15} +$	3119.0	(10.3)		$\nu_{18} +$	2856.0	(80.3)
			$\nu_{15} +$	3063.8	(5.8)		$\nu_{15} +$	3091.2	(5.4)		$\nu_{20} +$	2841.3	(7.4)
			$\nu_{18} +$	2875.8	(7.3)		$\nu_{16} +$	3069.5	(10.8)		$\nu_{67} +$	1313.6	(4.4)
			$\nu_{19} +$	2852.2	(9.7)		$\nu_{16} +$	3051.2	(10.1)		$\nu_{69} +$	1057.7	(10.0)

ν_{22} +	2877.3	(4.5)	ν_{19} +	2869.9	(4.7)
ν_{60} +	1412.2	(7.8)	ν_{19} +	2832.4	(6.5)
ν_{69} +	2827.9	(5.4)			

[a]Harmonic vibrational wavenumbers are scaled according to 0.9548 x + 27.9 for wavenumbers > 2000 cm^{-1} and 0.9804 x + 2.3 for wavenumbers < 2000 cm^{-1}, in which x is the harmonic vibrational wavenumbers. Harmonic IR intensities (in km mol^{-1}) are listed in parentheses. [b]Overtone and combination bands with the intensity > 4 km mol^{-1} are listed.

Table S3. Scaled Harmonic and Anharmonic Vibrational Wavenumbers and IR Intensities of 3- and 4-$HC_{14}H_{10}$ Predicted Using the B3LYP/6-311++G(d,p) Method

mode	sym.	3-$HC_{14}H_{10}$				4-$HC_{14}H_{10}$			
		calculation[a]				calculation[a]			
		harmonic		anharmonic		harmonic		anharmonic	
ν_1	a′	3081.9	(18.2)	3072.2	(7.3)	3079.5	(11.6)	3048.4	(4.3)
ν_2	a′	3068.9	(16.7)	3083.5	(9.2)	3067.5	(8.7)	3045.1	(15.3)
ν_3	a′	3062.8	(11.4)	3054.4	(5.1)	3067.1	(42.3)	3091.2	(40.1)
ν_4	a′	3060.6	(23.3)	3035.3	(12.4)	3058.5	(34.1)	3043.5	(60.2)
ν_5	a′	3053.5	(28.8)	3022.7	(3.8)	3055.3	(10.9)	3078.2	(4.4)
ν_6	a′	3052.6	(11.1)	3071.5	(23.5)	3051.9	(8.2)	3045.7	(35.1)
ν_7	a′	3043.0	(0.8)	2997.4	(0.0)	3043.0	(4.2)	3035.0	(4.7)
ν_8	a′	3040.0	(8.0)	3017.7	(16.3)	3040.4	(0.0)	3011.2	(0.9)
ν_9	a′	3031.3	(4.1)	2979.4	(3.7)	3039.4	(6.2)	3020.3	(11.5)
ν_{10}	a′	2822.4	(61.7)	2750.3	(42.3)	2835.5	(26.6)	2767.6	(8.5)
ν_{11}	a′	1631.9	(0.5)	1619.1	(0.2)	1615.9	(2.6)	1608.4	(1.1)
ν_{12}	a′	1610.5	(1.9)	1601.5	(2.0)	1603.3	(2.7)	1596.1	(3.0)
ν_{13}	a′	1565.2	(0.7)	1555.3	(0.1)	1559.4	(0.4)	1549.3	(0.0)
ν_{14}	a′	1528.7	(0.1)	1519.0	(0.0)	1536.8	(1.3)	1527.3	(0.4)
ν_{15}	a′	1499.0	(6.0)	1490.9	(0.8)	1514.7	(10.7)	1507.3	(6.9)
ν_{16}	a′	1479.8	(3.0)	1474.6	(2.2)	1461.3	(1.3)	1458.6	(1.6)
ν_{17}	a′	1436.1	(3.1)	1437.7	(0.8)	1432.8	(1.5)	1429.0	(1.1)
ν_{18}	a′	1423.6	(2.4)	1425.8	(2.6)	1418.5	(6.7)	1402.9	(4.8)
ν_{19}	a′	1414.7	(5.6)	1395.4	(1.2)	1415.0	(4.0)	1413.2	(4.0)
ν_{20}	a′	1410.9	(0.5)	1405.7	(6.1)	1390.9	(0.7)	1388.6	(0.0)
ν_{21}	a′	1357.8	(1.7)	1351.6	(0.3)	1382.0	(1.1)	1374.8	(0.2)
ν_{22}	a′	1338.3	(1.7)	1331.8	(0.4)	1358.4	(0.2)	1350.7	(0.4)
ν_{23}	a′	1318.9	(5.6)	1304.4	(5.1)	1321.2	(6.7)	1314.9	(4.0)
ν_{24}	a′	1285.8	(1.7)	1288.5	(0.6)	1291.5	(9.7)	1285.8	(8.3)
ν_{25}	a′	1266.4	(0.3)	1265.6	(0.4)	1265.1	(1.0)	1264.8	(0.4)
ν_{26}	a′	1239.8	(5.7)	1239.1	(3.3)	1214.0	(0.9)	1212.4	(0.7)
ν_{27}	a′	1215.8	(4.3)	1210.5	(1.9)	1207.1	(5.2)	1208.9	(4.6)
ν_{28}	a′	1190.6	(0.2)	1193.3	(0.1)	1179.1	(4.7)	1182.9	(1.4)
ν_{29}	a′	1161.5	(0.2)	1165.6	(0.1)	1160.6	(0.4)	1166.1	(0.8)
ν_{30}	a′	1155.3	(1.6)	1160.8	(1.3)	1155.2	(0.6)	1161.0	(0.5)
ν_{31}	a′	1141.3	(0.6)	1146.4	(0.2)	1146.8	(2.9)	1150.2	(1.7)
ν_{32}	a′	1104.0	(1.7)	1108.9	(1.3)	1102.3	(0.0)	1104.9	(0.1)

v_{33}	a′	1039.9	(5.5)	1041.1	(1.8)	1053.3	(1.9)	1054.6	(0.5)
v_{34}	a′	1019.2	(4.2)	1020.1	(2.3)	1033.2	(6.3)	1035.7	(5.4)
v_{35}	a′	982.0	(0.8)	988.0	(0.2)	986.6	(1.0)	990.8	(1.0)
v_{36}	a′	901.5	(10.0)	903.5	(8.8)	936.9	(8.0)	941.4	(1.1)
v_{37}	a′	853.5	(7.6)	856.1	(7.0)	846.2	(1.2)	849.6	(1.2)
v_{38}	a′	810.0	(1.9)	812.8	(1.9)	822.6	(0.2)	827.0	(0.3)
v_{39}	a′	704.5	(0.5)	708.2	(0.6)	703.9	(0.5)	708.1	(0.5)
v_{40}	a′	693.7	(0.9)	697.6	(0.7)	691.0	(1.1)	694.7	(0.9)
v_{41}	a′	607.4	(4.8)	610.9	(4.7)	594.8	(1.5)	598.5	(1.2)
v_{42}	a′	537.4	(0.1)	541.9	(0.1)	530.9	(0.1)	534.5	(0.1)
v_{43}	a′	485.0	(2.6)	488.2	(2.1)	486.8	(1.3)	490.4	(1.3)
v_{44}	a′	429.8	(0.4)	432.0	(0.2)	439.9	(0.9)	441.7	(0.9)
v_{45}	a′	397.2	(1.0)	397.2	(1.0)	399.0	(1.4)	400.8	(1.3)
v_{46}	a′	240.2	(0.5)	240.8	(0.4)	219.4	(4.4)	225.1	(4.1)
v_{47}	a″	2809.3	(17.3)	2747.3	(21.6)	2832.5	(14.8)	2777.5	(18.8)
v_{48}	a″	1166.9	(0.4)	1157.5	(0.2)	1185.2	(0.1)	1182.6	(0.2)
v_{49}	a″	971.2	(0.3)	966.9	(1.0)	971.1	(0.0)	995.0	(0.2)
v_{50}	a″	968.3	(0.0)	1004.0	(1.5)	961.9	(0.5)	970.5	(0.1)
v_{51}	a″	942.8	(0.8)	964.4	(0.0)	949.1	(1.0)	950.4	(2.2)
v_{52}	a″	934.0	(4.6)	932.5	(0.1)	943.5	(1.8)	955.1	(0.1)
v_{53}	a″	917.7	(8.6)	909.8	(13.6)	922.1	(1.7)	918.5	(1.1)
v_{54}	a″	855.7	(4.8)	880.1	(0.0)	852.8	(11.5)	868.2	(5.5)
v_{55}	a″	802.4	(65.4)	808.4	(69.7)	819.8	(40.1)	819.8	(37.2)
v_{56}	a″	780.9	(0.8)	793.7	(13.4)	777.4	(3.4)	794.2	(0.1)
v_{57}	a″	748.1	(16.7)	745.5	(10.8)	752.8	(27.3)	755.5	(10.4)
v_{58}	a″	717.8	(22.2)	720.1	(9.1)	736.5	(8.5)	734.9	(22.0)
v_{59}	a″	713.5	(1.3)	699.8	(12.9)	660.8	(56.3)	659.6	(51.2)
v_{60}	a″	653.3	(26.5)	634.5	(12.8)	643.3	(6.9)	644.2	(0.2)
v_{61}	a″	534.4	(8.9)	520.7	(10.0)	534.3	(1.9)	531.3	(4.6)
v_{62}	a″	507.6	(0.7)	499.3	(0.1)	523.6	(3.1)	518.6	(4.1)
v_{63}	a″	423.9	(0.9)	411.2	(2.8)	465.5	(1.5)	462.9	(1.6)
v_{64}	a″	413.5	(6.1)	411.6	(3.2)	412.3	(3.1)	415.4	(3.9)
v_{65}	a″	259.9	(1.3)	253.6	(2.0)	291.0	(3.4)	291.7	(2.9)
v_{66}	a″	232.1	(0.1)	219.5	(0.6)	229.9	(0.3)	233.0	(0.3)
v_{67}	a″	167.8	(3.1)	156.1	(2.2)	163.6	(1.0)	168.7	(0.7)
v_{68}	a″	100.4	(1.2)	97.9	(0.7)	116.4	(0.0)	117.1	(0.0)
v_{69}	a″	72.6	(0.1)	68.5	(0.3)	86.6	(0.1)	84.5	(0.0)
		overtone/combination mode[b]				overtone/combination mode[b]			

$2\nu_{19}$	2819.8	(16.8)	$2\nu_{18}$	2831.7	(5.2)
$2\nu_{50}$	2021.8	(7.6)	$2\nu_{49}$	2000.0	(5.2)
$\nu_{15} + \nu_{11}$	3114.3	(7.6)	$\nu_{14} + \nu_{12}$	3128.1	(8.4)
$\nu_{15} + \nu_{12}$	3089.3	(46.0)	$\nu_{14} + \nu_{13}$	3066.9	(4.0)
$\nu_{16} + \nu_{12}$	3058.3	(15.9)	$\nu_{15} + \nu_{11}$	3126.3	(6.1)
$\nu_{19} + \nu_{18}$	2814.4	(4.1)	$\nu_{15} + \nu_{12}$	3112.1	(7.6)
$\nu_{45} + \nu_{34}$	1417.7	(4.1)	$\nu_{16} + \nu_{12}$	3061.3	(9.8)
$\nu_{46} + \nu_{64}$	650.2	(6.0)	$\nu_{21} + \nu_{15}$	2885.8	(7.5)
$\nu_{66} + \nu_{39}$	927.5	(5.5)	$\nu_{67} + \nu_{41}$	762.3	(4.0)
			$\nu_{67} + \nu_{56}$	949.1	(5.1)
			$\nu_{68} + \nu_{39}$	825.1	(5.6)

[a]Harmonic vibrational wavenumbers are scaled according to 0.9548 x + 27.9 for wavenumbers > 2000 cm^{-1} and 0.9804 x + 2.3 for wavenumbers < 2000 cm^{-1}, in which x is the harmonic vibrational wavenumbers. Harmonic IR intensities (in km mol^{-1}) are listed in parentheses. [b]Overtone and combination wavenumbers with the intensity > 4 km mol^{-1} were listed.

Table S4. Scaled Harmonic Vibrational Wavenumbers and IR Intensities of 4a- and 8a-$HC_{14}H_{10}$ Predicted Using the B3LYP/6-311++G(d,p) Method

mode	sym.	4a-$HC_{14}H_{10}$ calculation[a]		8a-$HC_{14}H_{10}$ calculation[a]	
ν_1	a″	3085.1	(16.8)	3075.9	(18.1)
ν_2	a″	3074.6	(23.2)	3073.5	(23.9)
ν_3	a″	3071.1	(1.9)	3066.2	(13.1)
ν_4	a″	3065.4	(22.6)	3056.3	(32.1)
ν_5	a″	3057.5	(26.7)	3054.3	(25.8)
ν_6	a″	3053.2	(5.9)	3052.3	(2.1)
ν_7	a″	3044.9	(13.5)	3048.7	(2.8)
ν_8	a″	3042.5	(2.6)	3042.5	(5.6)
ν_9	a″	3037.4	(14.7)	3037.8	(2.9)
ν_{10}	a″	3033.9	(0.2)	3033.2	(2.5)
ν_{11}	a″	2669.8	(16.6)	2645.7	(24.3)
ν_{12}	a″	1601.3	(0.7)	1648.4	(0.2)
ν_{13}	a″	1592.7	(1.4)	1599.6	(0.9)
ν_{14}	a″	1578.5	(0.5)	1577.6	(1.9)
ν_{15}	a″	1528.9	(0.3)	1554.1	(1.2)
ν_{16}	a″	1490.8	(2.8)	1492.0	(1.8)
ν_{17}	a″	1475.9	(8.2)	1466.2	(13.0)
ν_{18}	a″	1449.8	(6.3)	1452.6	(6.4)
ν_{19}	a″	1424.9	(1.9)	1399.3	(1.3)
ν_{20}	a″	1393.6	(1.9)	1389.0	(1.3)
ν_{21}	a″	1349.8	(1.3)	1369.0	(0.5)
ν_{22}	a″	1312.1	(0.7)	1314.0	(1.5)
ν_{23}	a″	1306.1	(1.5)	1306.6	(2.9)
ν_{24}	a″	1283.5	(1.0)	1286.2	(1.6)
ν_{25}	a″	1249.8	(2.0)	1253.9	(11.2)
ν_{26}	a″	1226.6	(6.5)	1232.9	(5.2)
ν_{27}	a″	1211.8	(0.1)	1203.9	(1.8)
ν_{28}	a″	1194.4	(2.2)	1199.2	(3.1)
ν_{29}	a″	1178.8	(4.6)	1163.4	(0.1)
ν_{30}	a″	1167.9	(2.7)	1160.9	(0.1)
ν_{31}	a″	1161.1	(0.4)	1151.3	(0.5)
ν_{32}	a″	1136.6	(1.3)	1122.2	(0.8)

v_{33}	a″	1112.6	(0.5)	1117.0	(7.3)
v_{34}	a″	1104.8	(8.2)	1050.5	(0.4)
v_{35}	a″	1046.8	(3.5)	1043.2	(12.7)
v_{36}	a″	1027.7	(6.7)	1030.8	(5.1)
v_{37}	a″	991.3	(6.2)	997.3	(3.7)
v_{38}	a″	969.0	(0.0)	971.2	(4.4)
v_{39}	a″	959.9	(0.6)	970.1	(0.8)
v_{40}	a″	950.7	(0.9)	963.8	(1.3)
v_{41}	a″	946.1	(2.6)	951.7	(1.0)
v_{42}	a″	936.2	(3.6)	940.1	(2.5)
v_{43}	a″	918.1	(1.5)	918.7	(3.3)
v_{44}	a″	871.9	(3.3)	867.1	(2.5)
v_{45}	a″	848.9	(2.9)	858.7	(12.1)
v_{46}	a″	818.1	(3.3)	838.3	(6.5)
v_{47}	a″	806.2	(9.9)	788.3	(19.9)
v_{48}	a″	791.3	(49.2)	778.3	(20.6)
v_{49}	a″	766.7	(4.7)	756.9	(6.6)
v_{50}	a″	733.5	(13.8)	748.2	(31.1)
v_{51}	a″	711.3	(42.9)	695.5	(6.7)
v_{52}	a″	704.6	(12.3)	685.3	(19.4)
v_{53}	a″	676.3	(28.2)	675.9	(44.4)
v_{54}	a″	654.9	(1.8)	645.0	(5.7)
v_{55}	a″	593.1	(3.2)	603.3	(1.5)
v_{56}	a″	556.2	(0.6)	548.5	(2.9)
v_{57}	a″	535.6	(0.7)	533.9	(1.9)
v_{58}	a″	511.3	(4.7)	520.0	(1.4)
v_{59}	a″	479.8	(0.9)	497.8	(2.2)
v_{60}	a″	477.8	(3.8)	464.0	(1.7)
v_{61}	a″	423.0	(5.9)	424.3	(7.8)
v_{62}	a″	408.6	(3.0)	406.1	(1.4)
v_{63}	a″	382.9	(0.7)	368.1	(0.4)
v_{64}	a″	368.9	(0.7)	354.7	(1.4)
v_{65}	a″	245.4	(1.4)	255.3	(0.1)
v_{66}	a″	209.8	(0.7)	205.2	(0.1)
v_{67}	a″	203.3	(1.8)	177.5	(3.1)
v_{68}	a″	94.0	(0.3)	91.7	(0.2)

v_{69}	a″	50.4	(0.2)	70.2	(0.0)

[a]Harmonic vibrational wavenumbers are scaled according to 0.9548 x + 27.9 for wavenumbers > 2000 cm^{-1} and 0.9804 x + 2.3 for wavenumbers < 2000 cm^{-1}, in which x is the harmonic vibrational wavenumbers. Harmonic IR intensities (in km mol^{-1}) are listed in parentheses.

Table S5. Estimated Mixing Ratios of Each Species in the Electron-bombarded $C_{14}H_{10}$/*p*-H_2 Matrix Experiment and Integration Ranges

group	assignment	mixing ratio	integration range (cm^{-1})
parent	$C_{14}H_{10}$	31.55 ± 6.58 ppm	620−616, 740−733, 816−812, 867−864, 951−947, 1004−1001, 1043−1038, 1248−1241, 1463−1456, and 1507−1501
A	1-$HC_{14}H_{10}$	1.24 ± 0.08 ppm	653−657, 793−795, and 1276−1280
B	4-$HC_{14}H_{10}$	0.93 ± 0.32 ppm	658−663, 754−757, and 818−821
C	3-$HC_{14}H_{10}$	2.04 ± 0.20 ppm	649−653, 802−806, and 910−914
D	9-$HC_{14}H_{10}$	1.79 ± 0.66 ppm	750−752 and 1472−1475
E	2-$HC_{14}H_{10}$	2.00 ± 0.93 ppm	744−747, 787−789, and 1396−1399

Table S6. Vertical Excitation Wavelengths and Oscillator Strengths of Electronic Excitations of $C_{14}H_{10}$ and 9-H$C_{14}H_{10}$ Predicted Using the TD-B3LYP/6-311++G(d,p) Method

#	$C_{14}H_{10}$					9-H$C_{14}H_{10}$				
	λ[a]	f/10^{-2} [b]	assignment			λ[a]	f/10^{-2} [b]	assignment		
1	314	0.21	HOMO	→	LUMO+1	509	0.73	HOMO(α)	→	LUMO(α)
2	296	6.06	HOMO	→	LUMO	408	7.24	HOMO(β)	→	LUMO(β)
3	265	10.34	HOMO−1	→	LUMO	390	2.07	HOMO(α)	→	LUMO+1(α)
4	254	61.94	HOMO−1	→	LUMO+1	367	0.03	HOMO(α)	→	LUMO+1(α)
5	246	2.54	HOMO	→	LUMO+2	352	0.01	HOMO(α)	→	LUMO+5(α)
6	243	9.88	HOMO−2	→	LUMO+1	333	0.73	HOMO−1(β)	→	LUMO(β)
7	237	0.49	HOMO	→	LUMO+3	332	0.00	HOMO(α)	→	LUMO+2(α)
8	227	0.16	HOMO	→	LUMO+5	306	0.52	HOMO(α)	→	LUMO+5(α)
9	226	0.00	HOMO	→	LUMO+4	306	0.14	HOMO(α)	→	LUMO+3(α)
10	223	0.00	HOMO−1	→	LUMO+3	299	0.11	HOMO(α)	→	LUMO+4(α)
11	219	0.33	HOMO	→	LUMO+6	297	0.91	HOMO(α)	→	LUMO+8(α)
12	217	13.20	HOMO−2	→	LUMO	282	2.60	HOMO−2(β)	→	LUMO(β)
13						277	0.49	HOMO(β)	→	LUMO+2(β)
14						272	0.09	HOMO(α)	→	LUMO+6(α)
15						270	0.02	HOMO(α)	→	LUMO+7(α)
16						267	4.70	HOMO(β)	→	LUMO+1(β)
17						264	0.59	HOMO−2(α)	→	LUMO(α)
18						262	0.34	HOMO−1(α)	→	LUMO+1(α)
19						258	0.28	HOMO(α)	→	LUMO+9(α)
20						253	1.11	HOMO−1(α)	→	LUMO+1(α)
21						251	0.01	HOMO(α)	→	LUMO+10(α)
22						244	36.19	HOMO(α)	→	LUMO+8(α)

23	241	0.00	HOMO(α)	→	LUMO+11(α)
24	240	0.18	HOMO(α)	→	LUMO+12(α)
25	238	1.89	HOMO−2(β)	→	LUMO+1(β)
26	233	0.01	HOMO−4(β)	→	LUMO(β)
27	229	0.03	HOMO(β)	→	LUMO+3(β)
28	224	21.86	HOMO−1(α)	→	LUMO+1(α)
29	222	0.02	HOMO−1(α)	→	LUMO+2(α)
30	222	0.01	HOMO−5(β)	→	LUMO(β)
31	220	2.44	HOMO(β)	→	LUMO+6(β)
32	219	0.06	HOMO(β)	→	LUMO+4(β)
33	219	2.23	HOMO(α)	→	LUMO+14(α)
34	218	0.37	HOMO(β)	→	LUMO+5(β)
35	215	5.95	HOMO−3(α)	→	LUMO(α)
36	214	0.02	HOMO(α)	→	LUMO+13(α)
37	213	0.00	HOMO−1(α)	→	LUMO+3(α)
38	212	2.34	HOMO(α)	→	LUMO+15(α)
39	212	0.40	HOMO−1(α)	→	LUMO+4(α)
40	211	0.04	HOMO(α)	→	LUMO+17(α)

[a]Wavelength λ in nm. [b]Oscillator strength *f*.

Table S7. Vertical Excitation Wavelengths and Oscillator Strengths of Electronic Excitations of 1- and 2-$HC_{14}H_{10}$ Predicted Using the TD-B3LYP/6-311++G(d,p) Method

#	1-$HC_{14}H_{10}$					2-$HC_{14}H_{10}$				
	λ^a	$f/10^{-2\ b}$	assignment			λ^a	$f/10^{-2\ b}$	assignment		
1	566	0.42	HOMO(α)	→	LUMO(α)	596	0.05	HOMO(α)	→	LUMO(α)
2	417	1.55	HOMO(α)	→	LUMO+1(α)	446	4.72	HOMO(β)	→	LUMO(β)
3	414	13.19	HOMO(β)	→	LUMO(β)	415	0.35	HOMO(α)	→	LUMO+1(α)
4	384	0.23	HOMO(β)	→	LUMO+1(β)	405	0.07	HOMO(β)	→	LUMO+1 (β)
5	357	1.60	HOMO–1(β)	→	LUMO(β)	379	0.05	HOMO(α)	→	LUMO+3(α)
6	340	0.12	HOMO(α)	→	LUMO+2(α)	347	0.01	HOMO(α)	→	LUMO+2 (α)
7	335	2.34	HOMO(α)	→	LUMO+4(α)	333	7.53	HOMO–1(β)	→	LUMO(β)
8	309	0.00	HOMO(α)	→	LUMO+5(α)	314	2.80	HOMO–2(β)	→	LUMO(β)
9	304	0.18	HOMO(α)	→	LUMO+3(α)	312	0.01	HOMO(α)	→	LUMO+5(α)
10	298	2.64	HOMO–2(α)	→	LUMO(α)	310	0.01	HOMO(α)	→	LUMO+4(α)
11	295	0.73	HOMO–1(β)	→	LUMO+1(β)	302	0.05	HOMO(β)	→	LUMO+2(β)
12	287	4.21	HOMO–2(β)	→	LUMO(β)	283	0.05	HOMO(α)	→	LUMO+8(α)
13	285	0.26	HOMO–2(α)	→	LUMO+1(α)	279	0.38	HOMO(α)	→	LUMO+6(α)
14	280	1.48	HOMO(β)	→	LUMO+2(β)	278	0.00	HOMO(α)	→	LUMO+7(α)
15	275	0.47	HOMO(α)	→	LUMO+3(α)	271	0.05	HOMO(α)	→	LUMO+8(α)
16	274	0.02	HOMO(α)	→	LUMO+7(α)	269	0.11	HOMO–1(β)	→	LUMO+1(β)
17	273	1.30	HOMO–1(α)	→	LUMO(α)	261	59.43	HOMO(β)	→	LUMO+1(β)
18	268	0.76	HOMO–1(α)	→	LUMO+1(α)	257	0.03	HOMO(α)	→	LUMO+9(α)
19	265	0.14	HOMO(α)	→	LUMO+8(α)	257	7.57	HOMO(α)	→	LUMO+11(α)
20	256	0.00	HOMO(α)	→	LUMO+9(α)	254	9.04	HOMO(β)	→	LUMO+2(β)
21	251	0.69	HOMO–3(β)	→	LUMO(β)	251	9.33	HOMO–1(α)	→	LUMO+1(α)
22	245	0.00	HOMO(α)	→	LUMO+10(α)	248	0.08	HOMO(α)	→	LUMO+10(α)
23	242	0.04	HOMO(α)	→	LUMO+11(α)	245	0.01	HOMO(α)	→	LUMO+12(α)
24	236	0.18	HOMO(β)	→	LUMO+3(β)	244	8.93	HOMO–3(β)	→	LUMO(β)
25	234	0.59	HOMO–5(β)	→	LUMO(β)	244	0.05	HOMO–4(β)	→	LUMO(β)
26	234	0.00	HOMO–4(β)	→	LUMO(β)	243	0.15	HOMO(β)	→	LUMO+3(β)
27	229	0.03	HOMO(α)	→	LUMO+12(α)	241	4.36	HOMO(β)	→	LUMO+6(β)
28	227	0.08	HOMO–6(β)	→	LUMO(β)	238	3.31	HOMO–3(α)	→	LUMO(α)
29	226	0.26	HOMO–1(α)	→	LUMO+2(α)	236	3.19	HOMO–2(α)	→	LUMO+2(α)
30	226	0.00	HOMO(β)	→	LUMO+4(β)	232	0.01	HOMO(β)	→	LUMO+4(β)

31	224	0.10	HOMO(β)	→	LUMO+5(β)	231	0.24	HOMO–1(α)	→	LUMO+1(α)
32	223	7.74	HOMO(α)	→	LUMO+14(α)	231	0.06	HOMO(β)	→	LUMO+5(β)
33	223	1.46	HOMO–2(β)	→	LUMO+1(β)	227	3.91	HOMO(α)	→	LUMO+13(α)
34	221	39.70	HOMO–1(α)	→	LUMO+4(α)	226	0.22	HOMO–5(β)	→	LUMO(β)
35	220	0.01	HOMO(α)	→	LUMO+13(α)	222	0.03	HOMO(α)	→	LUMO+14(α)
36	219	16.50	HOMO–1(α)	→	LUMO+14(α)	222	0.80	HOMO(α)	→	LUMO+13(α)
37	218	22.95	HOMO(α)	→	LUMO+14(α)	221	0.00	HOMO–1(α)	→	LUMO+4(α)
38	217	0.04	HOMO–1(α)	→	LUMO+3(α)	220	0.04	HOMO–1(α)	→	LUMO+5(α)
39	216	1.92	HOMO(α)	→	LUMO+16(α)	219	0.57	HOMO(α)	→	LUMO+15(α)
40	215	0.06	HOMO–1(α)	→	LUMO+5(α)	219	0.09	HOMO–6(β)	→	LUMO(β)

[a]Wavelength λ in nm. [b]Oscillator strength *f*.

Table S8. Vertical Excitation Wavelengths and Oscillator Strengths of Electronic Excitations of 3- and 4-$HC_{14}H_{10}$ Predicted Using the TD-B3LYP/6-311++G(d,p) Method

#	3-$HC_{14}H_{10}$					4-$HC_{14}H_{10}$				
	λ[a]	f/10^{-2} [b]	assignment			λ[a]	f/10^{-2} [b]	assignment		
1	569	0.16	HOMO(α)	→	LUMO(α)	554	0.25	HOMO(α)	→	LUMO(α)
2	474	0.19	HOMO(α)	→	LUMO+1(α)	435	3.74	HOMO(β)	→	LUMO(β)
3	410	4.24	HOMO(β)	→	LUMO(β)	411	1.89	HOMO(α)	→	LUMO+1(α)
4	384	14.08	HOMO−1(β)	→	LUMO(β)	394	0.20	HOMO(β)	→	LUMO+1(β)
5	362	0.22	HOMO(β)	→	LUMO+1(β)	352	15.60	HOMO−1(β)	→	LUMO(β)
6	348	0.05	HOMO(α)	→	LUMO+2(α)	335	0.06	HOMO(α)	→	LUMO+2(α)
7	338	0.08	HOMO(α)	→	LUMO+5(α)	329	0.58	HOMO(α)	→	LUMO+5(α)
8	316	0.08	HOMO(α)	→	LUMO+4(α)	305	0.01	HOMO(α)	→	LUMO+4(α)
9	312	0.07	HOMO(α)	→	LUMO+3(α)	300	0.17	HOMO(α)	→	LUMO+5(α)
10	300	0.13	HOMO−3(β)	→	LUMO(β)	299	0.00	HOMO(α)	→	LUMO+3(α)
11	296	0.08	HOMO(α)	→	LUMO+5(α)	290	0.69	HOMO(α)	→	LUMO+5(α)
12	291	0.37	HOMO−1(α)	→	LUMO+1(α)	283	2.62	HOMO−2(β)	→	LUMO(β)
13	282	0.63	HOMO−2(β)	→	LUMO(β)	277	2.53	HOMO(β)	→	LUMO+2(β)
14	281	0.01	HOMO(α)	→	LUMO+6(α)	272	19.53	HOMO(β)	→	LUMO+1(β)
15	279	0.16	HOMO(α)	→	LUMO+7(α)	272	0.19	HOMO(α)	→	LUMO+6(α)
16	274	0.86	HOMO(α)	→	LUMO+8(α)	270	1.34	HOMO(β)	→	LUMO+2(β)
17	270	2.07	HOMO−1(β)	→	LUMO+1(β)	269	0.32	HOMO(α)	→	LUMO+7(α)
18	268	0.53	HOMO(α)	→	LUMO+9(α)	266	0.01	HOMO−2(β)	→	LUMO(β)
19	264	0.17	HOMO−2(β)	→	LUMO+1(β)	262	0.09	HOMO(α)	→	LUMO+8(α)
20	256	5.11	HOMO(α)	→	LUMO+9(α)	255	0.16	HOMO−1(β)	→	LUMO+2(β)
21	255	0.03	HOMO(α)	→	LUMO+12(α)	254	0.00	HOMO(α)	→	LUMO+9(α)
22	251	0.05	HOMO(α)	→	LUMO+11(α)	243	2.84	HOMO(α)	→	LUMO+12(α)
23	248	0.23	HOMO(α)	→	LUMO+10(α)	241	0.03	HOMO(α)	→	LUMO+10(α)
24	248	17.29	HOMO−2(α)	→	LUMO(α)	240	0.30	HOMO(α)	→	LUMO+11(α)
25	240	0.01	HOMO−4(β)	→	LUMO(β)	239	0.31	HOMO(β)	→	LUMO+3(β)
26	233	1.22	HOMO(α)	→	LUMO+13(α)	236	0.02	HOMO−4(β)	→	LUMO(β)
27	231	14.49	HOMO−3(β)	→	LUMO+1(β)	234	1.00	HOMO(α)	→	LUMO+12(α)
28	231	0.00	HOMO(β)	→	LUMO+3(β)	230	0.26	HOMO−1(α)	→	LUMO+2(α)
29	230	57.80	HOMO−2(α)	→	LUMO+1(α)	228	0.06	HOMO(β)	→	LUMO+4(β)
30	226	0.02	HOMO−5(β)	→	LUMO(β)	227	0.05	HOMO−5(β)	→	LUMO(β)

31	224	9.28	HOMO−1(α)	→	LUMO+1(α)	226	0.81	HOMO(α)	→	LUMO+13(α)
32	224	0.00	HOMO−1(β)	→	LUMO+3(β)	225	0.08	HOMO(β)	→	LUMO+5(β)
33	223	5.51	HOMO(α)	→	LUMO+13(α)	222	0.44	HOMO(β)	→	LUMO+6(β)
34	222	0.00	HOMO(α)	→	LUMO+14(α)	221	4.65	HOMO−1(α)	→	LUMO+5(α)
35	219	0.35	HOMO(β)	→	LUMO+4(β)	220	0.04	HOMO−1(α)	→	LUMO+3(α)
36	219	0.08	HOMO−1(α)	→	LUMO+2(α)	217	0.08	HOMO−1(α)	→	LUMO+4(α)
37	218	9.28	HOMO(α)	→	LUMO+15(α)	214	0.00	HOMO(α)	→	LUMO+14(α)
38	217	0.14	HOMO(α)	→	LUMO+16(α)	214	48.70	HOMO−1(β)	→	LUMO+1(β)
39	216	0.08	HOMO(β)	→	LUMO+5(β)	213	0.00	HOMO(α)	→	LUMO+15(α)
40	215	0.13	HOMO−2(α)	→	LUMO+2(α)	212	1.88	HOMO−6(β)	→	LUMO(β)

[a]Wavenumber λ in nm. [b]Oscillator strength *f*.

Table S9. Vertical Excitation Wavelengths and Oscillator Strengths of Electronic Excitations of 4a- and 8a-$HC_{14}H_{10}$ Predicted Using the TD-B3LYP/6-311++G(d,p) Method

#	4a-$HC_{14}H_{10}$					8a-$HC_{14}H_{10}$				
	λ^a	$f/10^{-2\ b}$	assignment			λ^a	$f/10^{-2\ b}$	assignment		
1	616	0.11	HOMO(α)	→	LUMO(α)	571	0.40	HOMO(α)	→	LUMO(α)
2	453	13.55	HOMO(β)	→	LUMO(β)	482	0.47	HOMO(α)	→	LUMO+1(α)
3	422	1.42	HOMO(α)	→	LUMO+2(α)	448	7.87	HOMO(β)	→	LUMO(β)
4	393	2.08	HOMO(α)	→	LUMO+1(α)	392	3.44	HOMO−1(α)	→	LUMO(α)
5	369	0.90	HOMO−1(β)	→	LUMO(β)	382	8.29	HOMO−1(β)	→	LUMO(β)
6	353	1.79	HOMO−1(β)	→	LUMO(β)	350	0.00	HOMO(α)	→	LUMO+5(α)
7	335	0.63	HOMO(α)	→	LUMO+3(α)	336	0.31	HOMO(α)	→	LUMO+2(α)
8	326	3.37	HOMO−2(β)	→	LUMO(β)	322	0.01	HOMO(α)	→	LUMO+5(α)
9	317	0.18	HOMO−1(α)	→	LUMO+2(α)	308	0.20	HOMO(α)	→	LUMO+4(α)
10	311	0.12	HOMO(α)	→	LUMO+5(α)	305	0.02	HOMO(α)	→	LUMO+3(α)
11	307	0.28	HOMO(α)	→	LUMO+4(α)	300	0.31	HOMO(α)	→	LUMO+5(α)
12	291	0.30	HOMO(α)	→	LUMO+6(α)	295	0.35	HOMO(α)	→	LUMO+6(α)
13	285	0.02	HOMO−1(β)	→	LUMO+2(β)	290	0.23	HOMO−2(β)	→	LUMO(β)
14	276	0.17	HOMO(β)	→	LUMO+2(β)	279	1.09	HOMO−1(α)	→	LUMO+1(α)
15	274	0.31	HOMO(α)	→	LUMO+7(α)	277	0.44	HOMO−1(β)	→	LUMO+1(β)
16	272	0.11	HOMO(α)	→	LUMO+7(α)	274	0.53	HOMO(α)	→	LUMO+7(α)
17	269	17.30	HOMO−1(α)	→	LUMO(α)	274	0.58	HOMO(α)	→	LUMO+7(α)
18	264	0.30	HOMO−2(α)	→	LUMO(α)	271	0.37	HOMO(α)	→	LUMO+8(α)
19	263	0.20	HOMO−3(β)	→	LUMO(β)	264	11.19	HOMO−1(α)	→	LUMO(α)
20	256	0.70	HOMO−1(α)	→	LUMO+1(α)	263	6.90	HOMO(β)	→	LUMO+1(β)
21	255	2.40	HOMO−4(β)	→	LUMO(β)	253	0.16	HOMO−3(β)	→	LUMO(β)
22	247	0.42	HOMO(α)	→	LUMO+9(α)	249	0.11	HOMO(α)	→	LUMO+9(α)
23	246	0.97	HOMO(α)	→	LUMO+10(α)	246	0.44	HOMO(α)	→	LUMO+10(α)
24	245	0.84	HOMO(α)	→	LUMO+10(α)	245	0.12	HOMO(α)	→	LUMO+9(α)
25	242	3.01	HOMO(α)	→	LUMO+12(α)	243	5.70	HOMO−4(β)	→	LUMO(β)
26	242	0.96	HOMO(α)	→	LUMO+12(α)	241	0.72	HOMO(α)	→	LUMO+12(α)
27	240	0.97	HOMO−2(β)	→	LUMO+1(β)	237	4.77	HOMO(β)	→	LUMO+3(β)
28	237	0.59	HOMO(β)	→	LUMO+3(β)	236	1.24	HOMO−5(β)	→	LUMO(β)
29	236	0.10	HOMO−5(β)	→	LUMO(β)	236	9.27	HOMO(β)	→	LUMO+3(β)
30	232	7.93	HOMO−1(α)	→	LUMO+2(α)	231	20.83	HOMO−1(α)	→	LUMO+2(α)

31	229	1.80	HOMO−5(β)	→	LUMO(β)	230	14.29	HOMO−1(α)	→	LUMO+2(α)
32	228	0.25	HOMO−1(α)	→	LUMO+3(α)	227	0.54	HOMO−6(β)	→	LUMO(β)
33	227	0.20	HOMO(β)	→	LUMO+6(β)	225	0.96	HOMO(α)	→	LUMO+13(α)
34	225	0.34	HOMO(β)	→	LUMO+5(β)	225	0.16	HOMO(β)	→	LUMO+5(β)
35	225	0.09	HOMO(β)	→	LUMO+5(β)	224	0.25	HOMO(β)	→	LUMO+4(β)
36	224	2.78	HOMO(α)	→	LUMO+14(α)	223	1.10	HOMO−1(α)	→	LUMO+5(α)
37	221	0.11	HOMO−6(β)	→	LUMO(β)	222	0.14	HOMO−2(β)	→	LUMO+1(β)
38	220	0.34	HOMO−6(β)	→	LUMO(β)	222	2.13	HOMO(α)	→	LUMO+14(α)
39	218	0.55	HOMO(α)	→	LUMO+15(α)	219	0.17	HOMO−1(α)	→	LUMO+3(α)
40	218	0.69	HOMO−1(α)	→	LUMO+5(α)	218	0.34	HOMO−1(α)	→	LUMO+4(α)

[a]Wavenumber λ in nm. [b]Oscillator strength *f*.

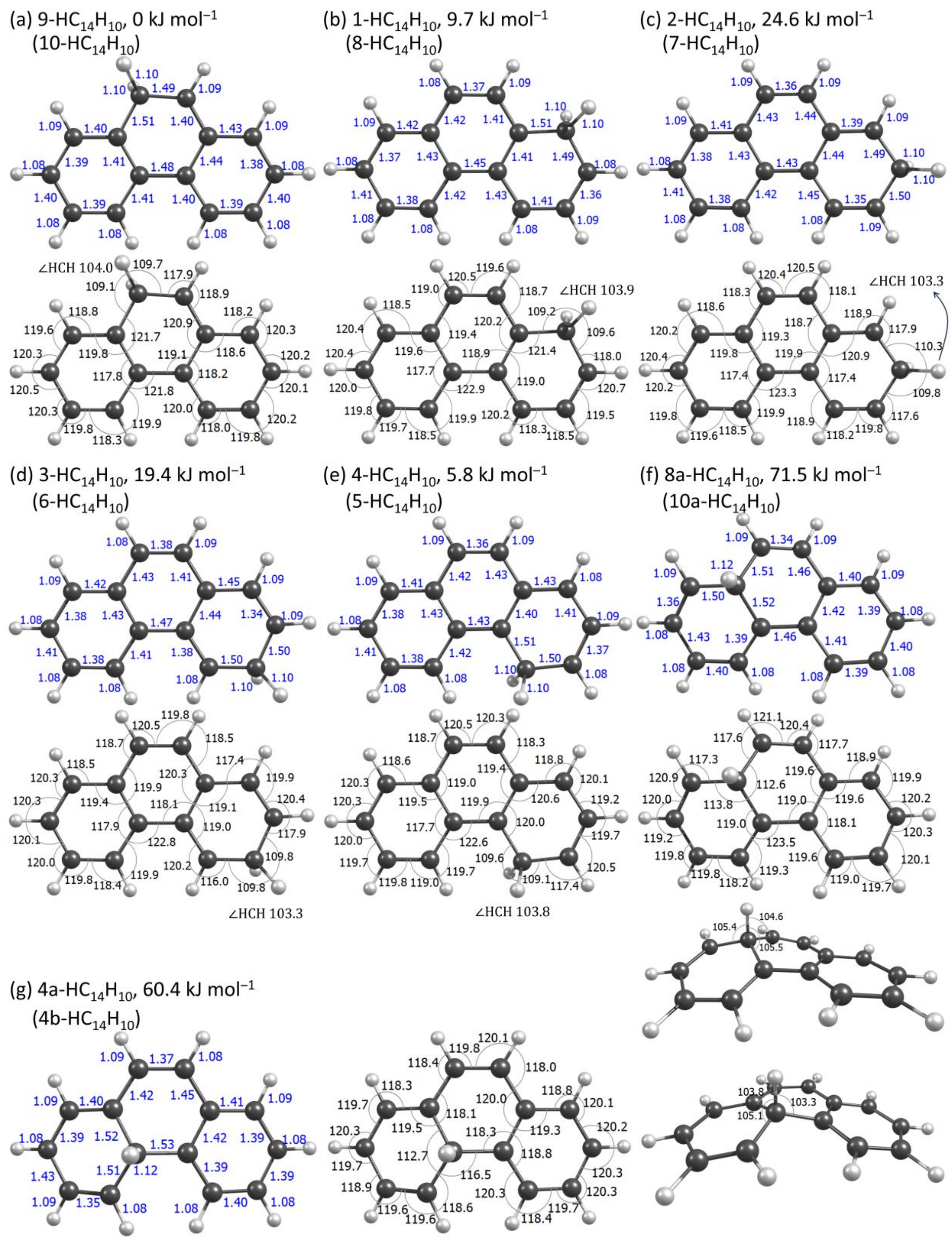

Figure S1. Geometries and relative energies of seven isomers of $HC_{14}H_{10}$. Geometries were optimized with the B3LYP/6-311++G(d,p) method and their energies were calculated with the CCSD(T)/6-311++G(d,p)//B3LYP/6-311++G(d,p) method. Zero-point vibrational energies (ZPVE) were corrected according to harmonic vibrational wavenumbers calculated with the B3LYP/6-311++G(d,p) method. Bond lengths (blue) are in Å and bond angles (black) are in degrees.

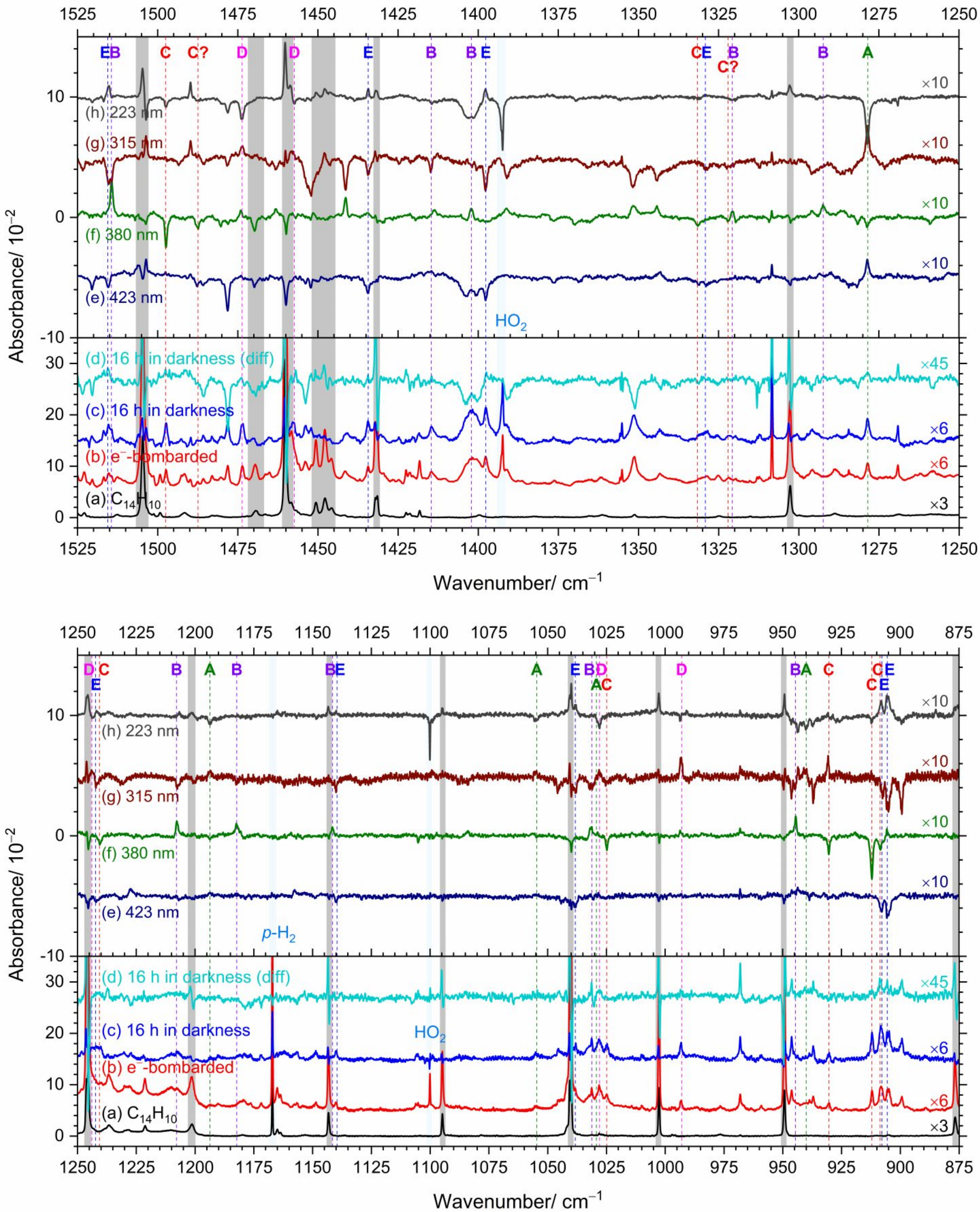
Absorbance/ 10^{-2}
Wavenumber/ cm^{-1}
(h) 223 nm
(g) 315 nm
(f) 380 nm
(e) 423 nm
(d) 16 h in darkness (diff)
(c) 16 h in darkness
(b) e$^-$-bombarded
(a) $C_{14}H_{10}$
HO$_2$
p-H$_2$
×10
×45
×6
×3

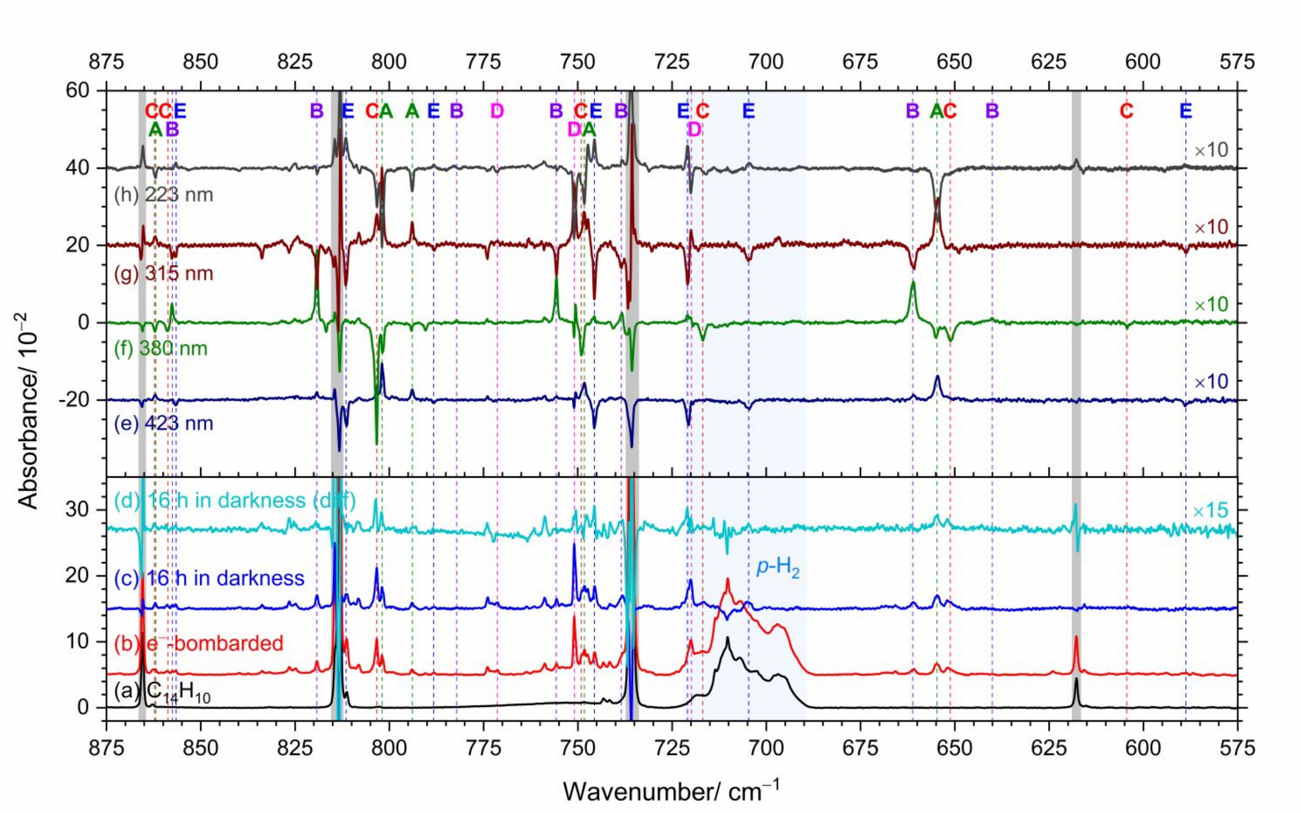

Figure S2. Infrared spectra (in the region 575–1525 cm^{-1}) of an electron-bombarded $C_{14}H_{10}$/p-H_2 matrix after each experimental step. (a) $C_{14}H_{10}$/p-H_2 matrix without electron bombardment. (b) Electron-bombarded $C_{14}H_{10}$/p-H_2 matrix. (c) Spectrum recorded after maintenance of the matrix in darkness for 16 h, with bands of $C_{14}H_{10}$ and p-H_2 subtracted. (d) Difference spectrum measured after maintenance of the matrix in darkness for 16 h. Difference spectra after secondary irradiation at 423 nm (e), 380 nm (f), 315 nm (g), and 223 nm (h); each irradiation step is 20 min. Bands in groups A, B, C, D, and E are indicated with color-coded labels and dashed lines. Spectral regions subjected to interference from the intense absorption of $C_{14}H_{10}$ are shaded gray, and those from p-H_2 and HO_2 are shaded light blue. Baselines are shifted for clarity.

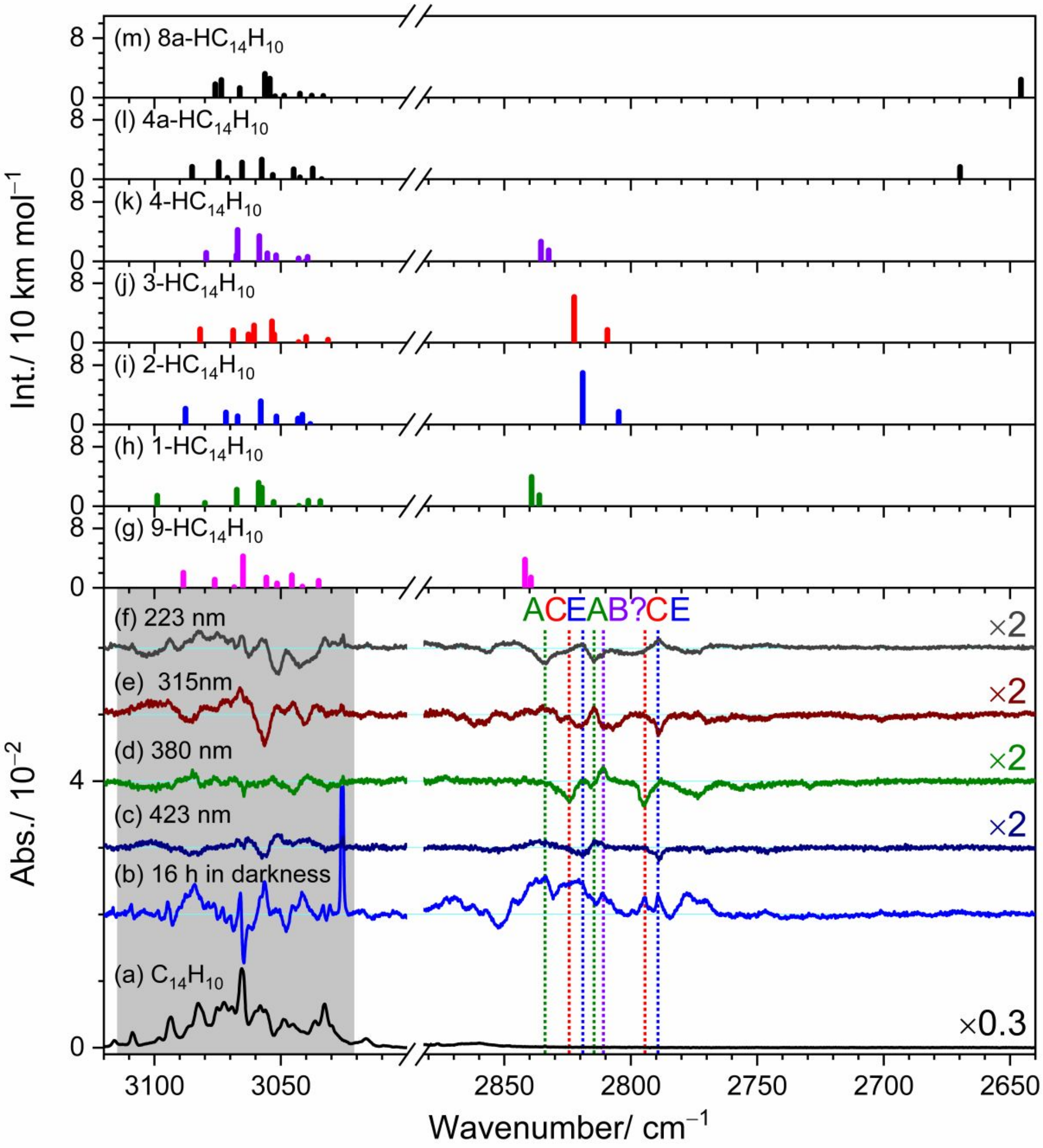

Figure S3. Comparison of observed IR spectra in the region 2640–3120 cm^{-1} with stick spectra of seven isomers of $HC_{14}H_{10}$. The stick spectra are based on scaled harmonic vibrational wavenumbers and IR intensities calculated with the B3LYP/6-311++G(d,p) method. (a) $C_{14}H_{10}$/*p*-H_2 matrix without electron bombardment. (b) Spectrum recorded after maintenance of the matrix in darkness for 16 h, with bands of $C_{14}H_{10}$ and *p*-H_2 subtracted. Difference spectra measured after secondary irradiation at 423 nm (c), 380 nm (d), 315 nm (e), and 223 nm (f); each irradiation step is 20 min. Bands in groups A, B, C, D, and E are indicated with color-coded labels and dashed lines. Spectral regions subjected to interference from the intense absorption of $C_{14}H_{10}$ are shaded gray. Baselines are shifted for clarity.

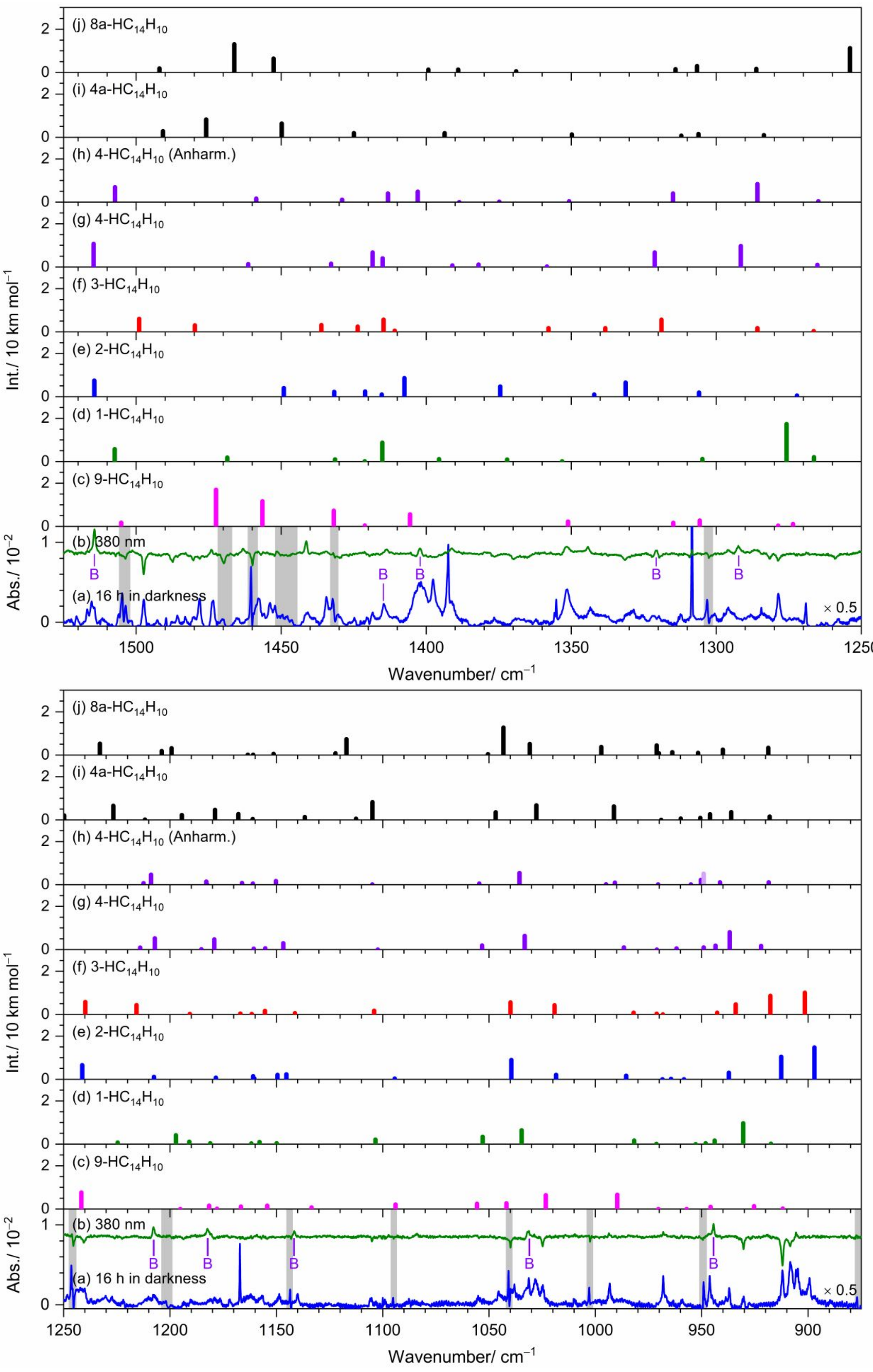

(j) 8a-$HC_{14}H_{10}$
(i) 4a-$HC_{14}H_{10}$
(h) 4-$HC_{14}H_{10}$ (Anharm.)
(g) 4-$HC_{14}H_{10}$
(f) 3-$HC_{14}H_{10}$
(e) 2-$HC_{14}H_{10}$
(d) 1-$HC_{14}H_{10}$
(c) 9-$HC_{14}H_{10}$
(b) 380 nm
(a) 16 h in darkness
B
× 0.5
Int./ 10 km mol^{-1}
Abs./ 10^{-2}
Wavenumber/ cm^{-1}

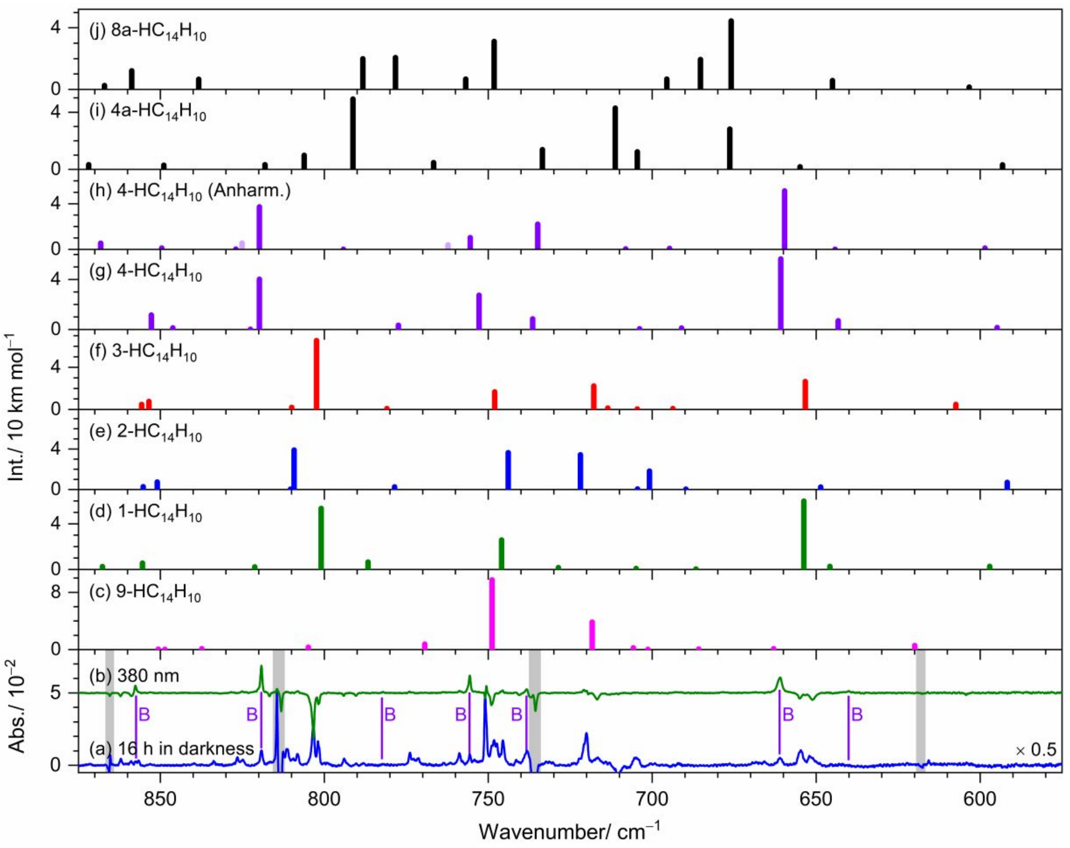

Figure S4. Comparison of bands in group B in the region 575−1525 cm^{-1} with stick spectra of seven isomers of $HC_{14}H_{10}$. (a) and (b) are experimental spectra taken from Figure S2. (a) Spectrum measured after maintenance of the matrix in darkness for 16 h. (b) Difference spectra after secondary irradiation at 380 nm for 20 min. Bands in group B are indicated with violet labels and lines. Spectral regions subjected to interference from the intense absorption of $C_{14}H_{10}$ are shaded gray. Baselines are shifted for clarity. The stick spectra (c)−(g), (i), and (j) are based on scaled harmonic vibrational wavenumbers and IR intensities calculated with the B3LYP/6-311++G(d,p) method. The anharmonic vibrational stick spectrum of 4-$HC_{14}H_{10}$ is shown in trace (h), with combination and overtone bands in light color.

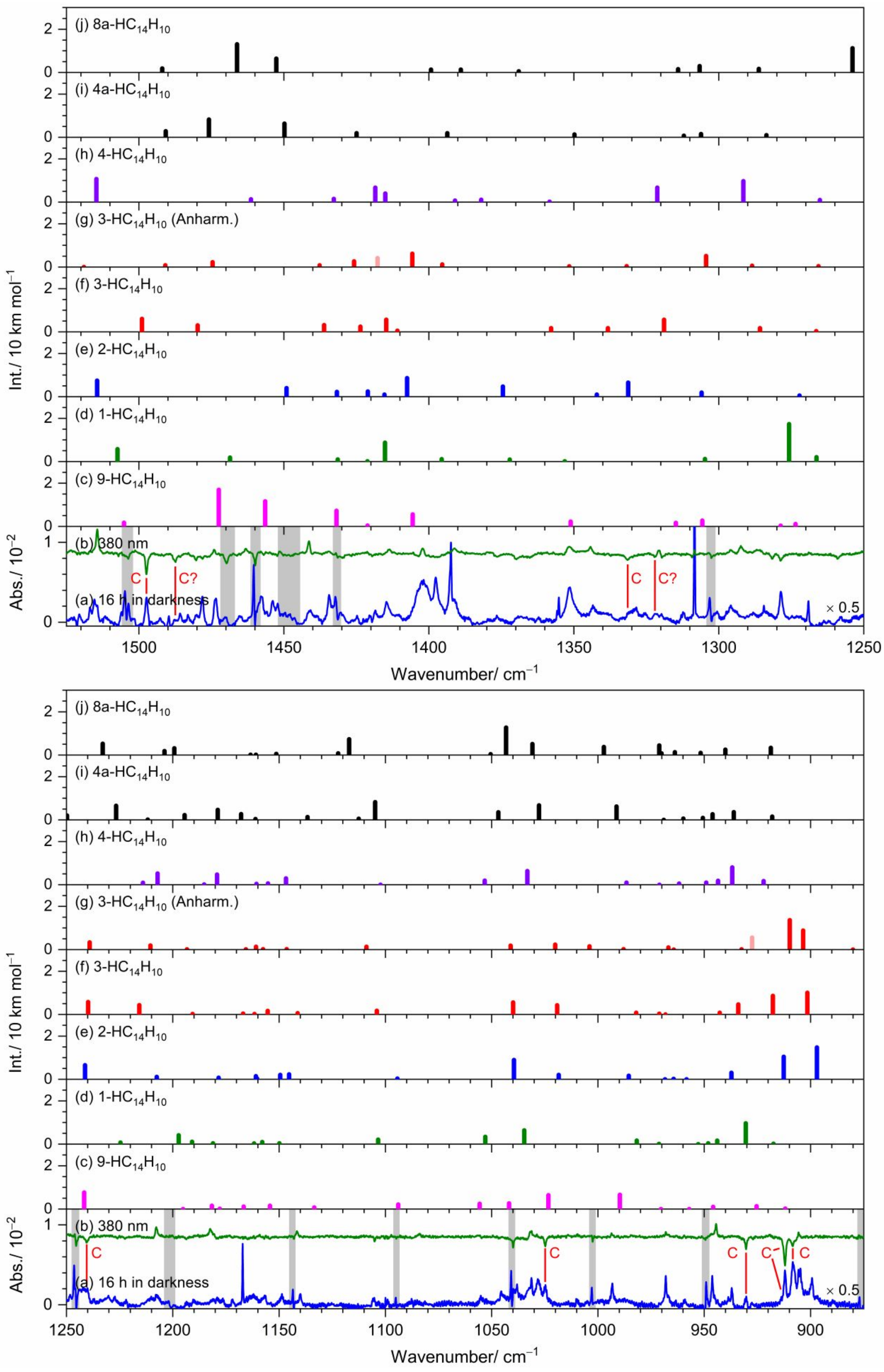

(j) 8a-$HC_{14}H_{10}$
(i) 4a-$HC_{14}H_{10}$
(h) 4-$HC_{14}H_{10}$
(g) 3-$HC_{14}H_{10}$ (Anharm.)
(f) 3-$HC_{14}H_{10}$
(e) 2-$HC_{14}H_{10}$
(d) 1-$HC_{14}H_{10}$
(c) 9-$HC_{14}H_{10}$
(b) 380 nm
(a) 16 h in darkness
Int./ 10 km mol^{-1}
Abs./ 10^{-2}
Wavenumber/ cm^{-1}
× 0.5
C
C?

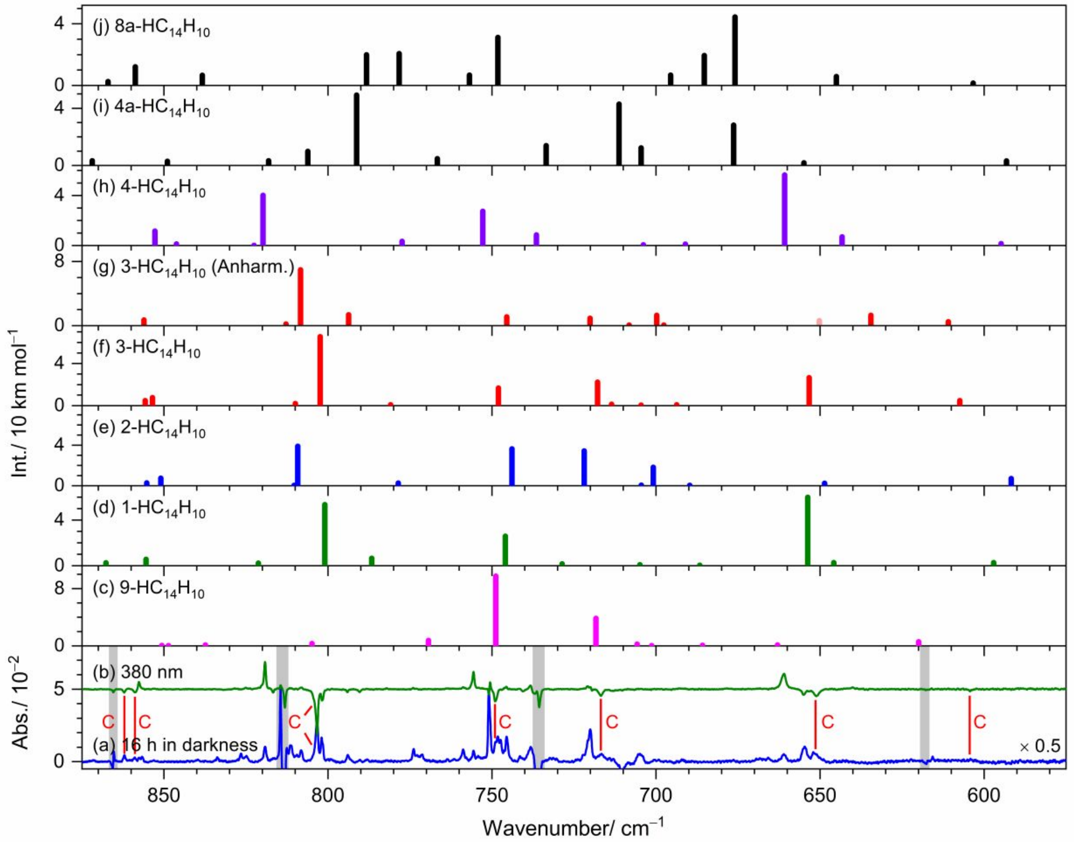

Figure S5. Comparison of bands in group C in the region 575−1525 cm^{-1} with stick spectra of seven isomers of $HC_{14}H_{10}$. (a) and (b) are experimental spectra taken from Figure S2. (a) Spectrum measured after maintenance of the matrix in darkness for 16 h. (b) Difference spectra after secondary irradiation at 380 nm for 20 min. Bands in group C are indicated with red labels and lines. Spectral regions subjected to interference from the intense absorption of $C_{14}H_{10}$ are shaded gray. Baselines are shifted for clarity. The stick spectra (c)−(f), and (h)−(j) are based on scaled harmonic vibrational wavenumbers and IR intensities calculated with the B3LYP/6-311++G(d,p) method. The anharmonic vibrational stick spectrum of 3-$HC_{14}H_{10}$ is shown in trace (g), with combination and overtone bands in light color.

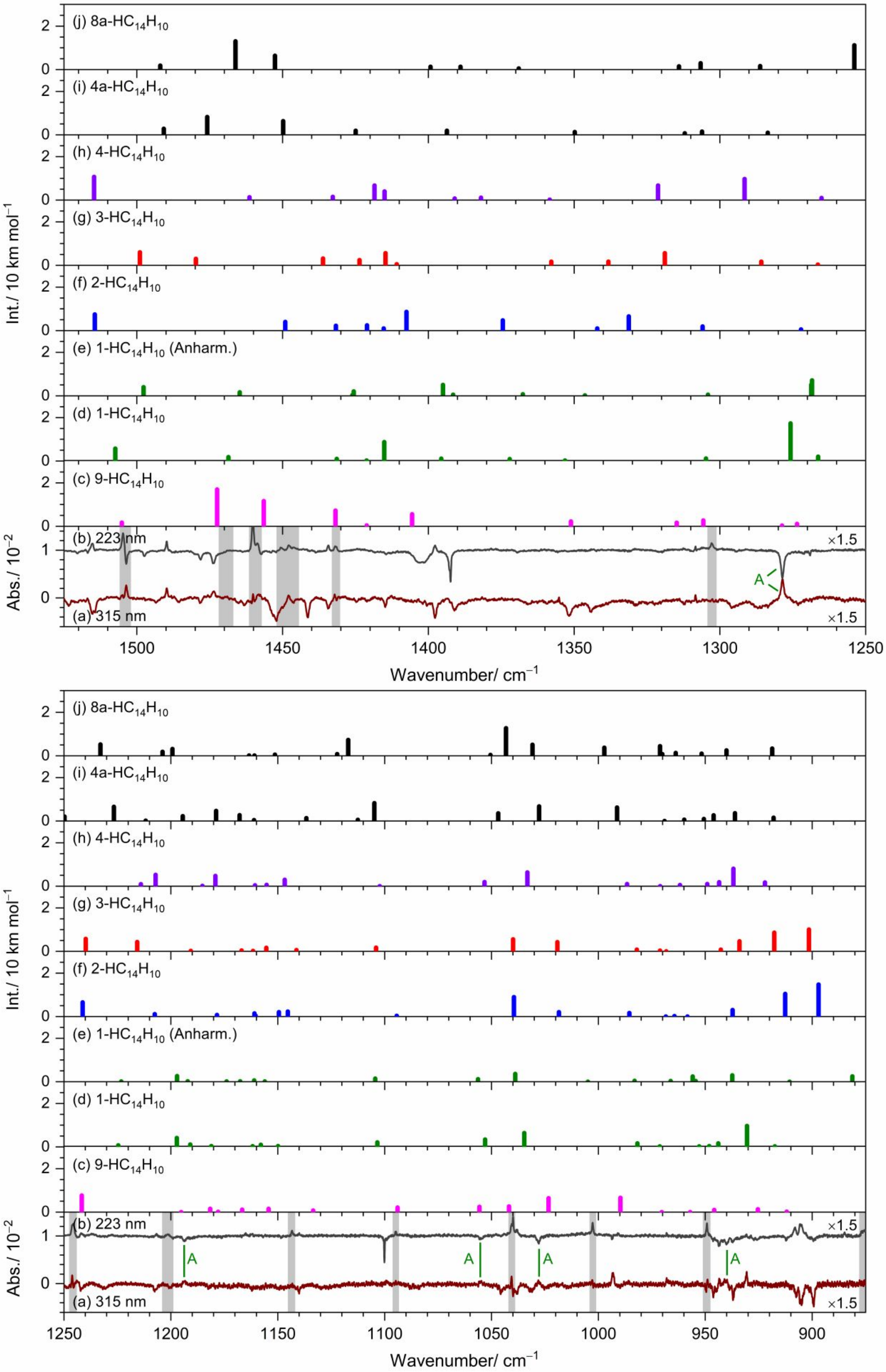

(j) 8a-HC14H10
(i) 4a-HC14H10
(h) 4-HC14H10
(g) 3-HC14H10
(f) 2-HC14H10
(e) 1-HC14H10 (Anharm.)
(d) 1-HC14H10
(c) 9-HC14H10
(b) 223 nm
(a) 315 nm
×1.5
A
Int./ 10 km mol−1
Abs./ 10−2
Wavenumber/ cm−1

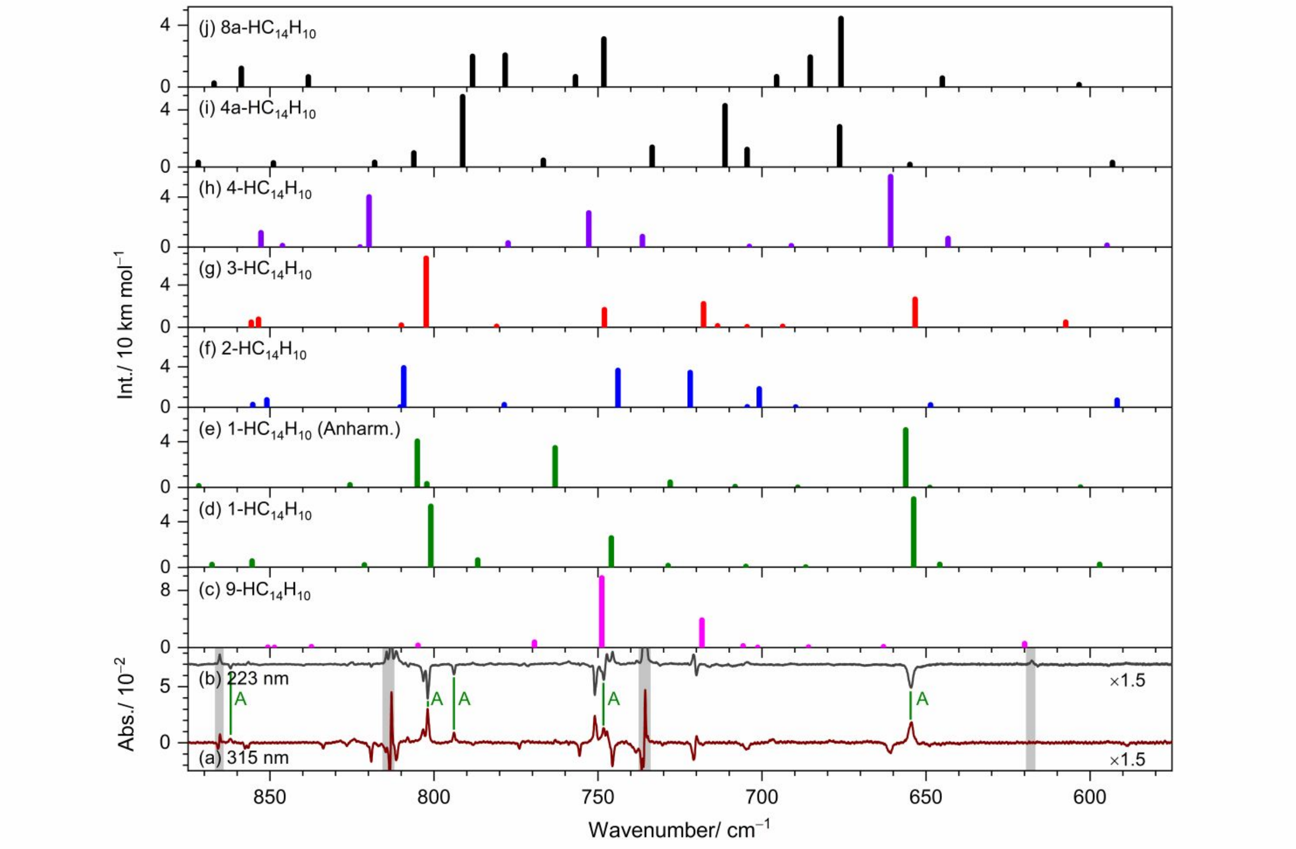

Figure S6. Comparison of bands in group A in the region 575−1525 cm^{-1} with stick spectra of seven isomers of $HC_{14}H_{10}$. Difference spectra after secondary irradiation at 315 nm (a) and 223 nm (b) are experimental spectra taken from Figure S2. Bands in group A are indicated with green labels and lines. Spectral regions subjected to interference from the intense absorption of $C_{14}H_{10}$ are shaded gray. Baselines are shifted for clarity. The stick spectra (c), (d), and (f)−(j) are based on scaled harmonic vibrational wavenumbers and IR intensities calculated with the B3LYP/6-311++G(d,p) method. The anharmonic vibrational stick spectrum of 1-$HC_{14}H_{10}$ is shown in trace (e), with combination and overtone bands in light color.

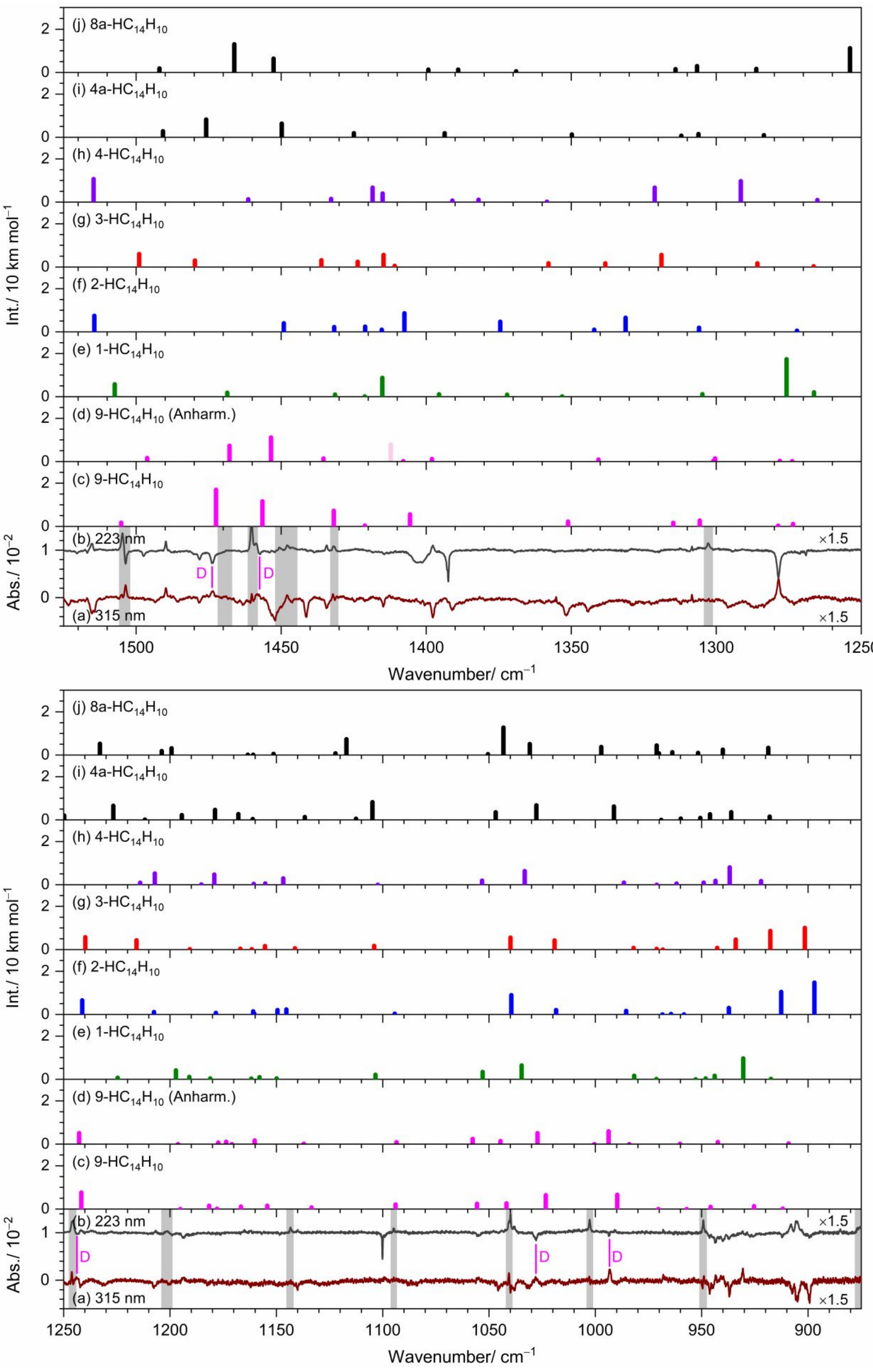
(j) 8a-HC14H10
(i) 4a-HC14H10
(h) 4-HC14H10
(g) 3-HC14H10
(f) 2-HC14H10
(e) 1-HC14H10
(d) 9-HC14H10 (Anharm.)
(c) 9-HC14H10
(b) 223 nm
(a) 315 nm
×1.5
D
Int./ 10 km mol−1
Abs./ 10−2
Wavenumber/ cm−1
1500
1450
1400
1350
1300
1250
1200
1150
1100
1050
1000
950
900

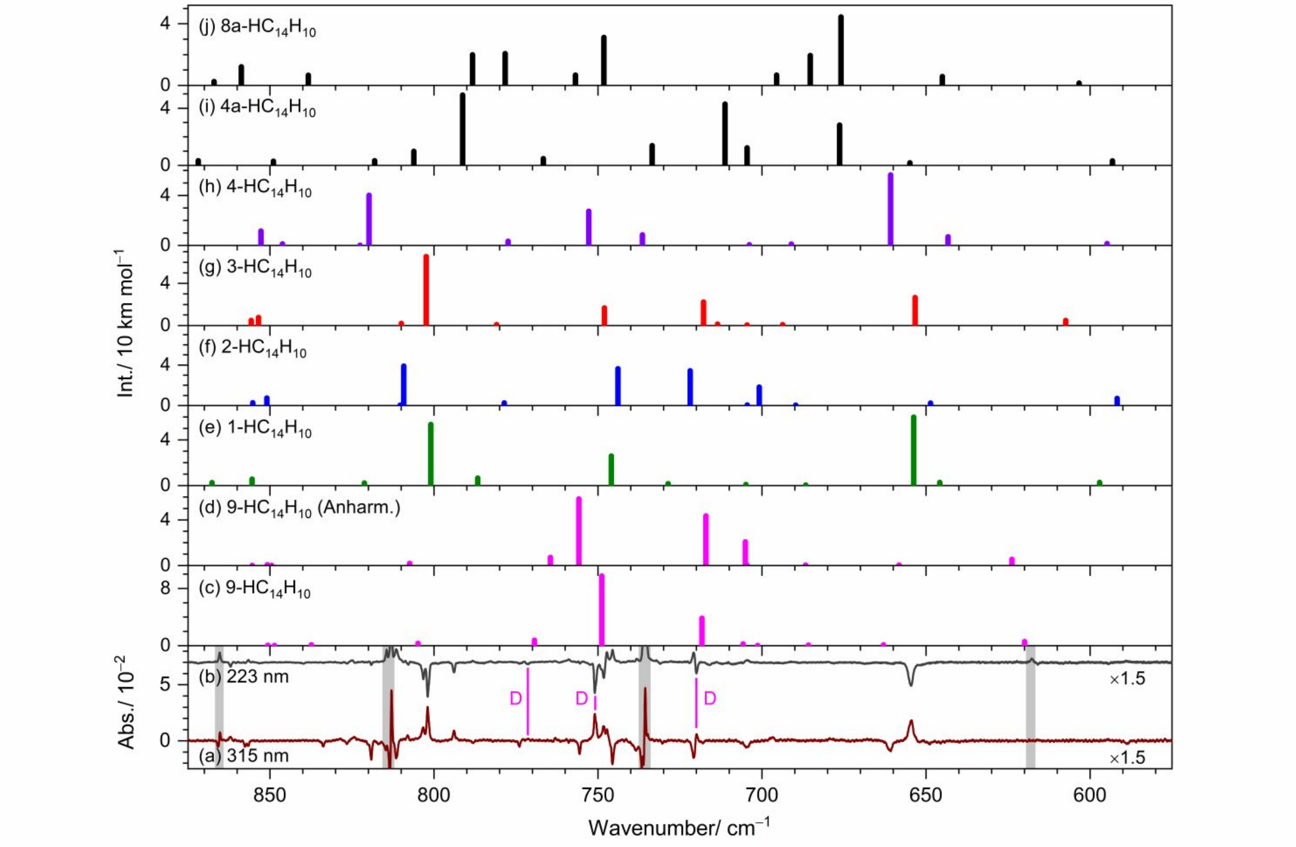

Figure S7. Comparison of bands in group D in the region 575−1525 cm^{-1} with stick spectra of seven isomers of $HC_{14}H_{10}$. Difference spectra after secondary irradiation at 315 nm (a) and 223 nm (b) are experimental spectra taken from Figure S2. Bands in group D are indicated with pink labels and lines. Spectral regions subjected to interference from the intense absorption of $C_{14}H_{10}$ are shaded gray. Baselines are shifted for clarity. The stick spectra (c) and (e)−(j) are based on scaled harmonic vibrational wavenumbers and IR intensities calculated with the B3LYP/6-311++G(d,p) method. The anharmonic vibrational stick spectrum of 9-$HC_{14}H_{10}$ is shown in trace (d), with combination and overtone bands in light color.

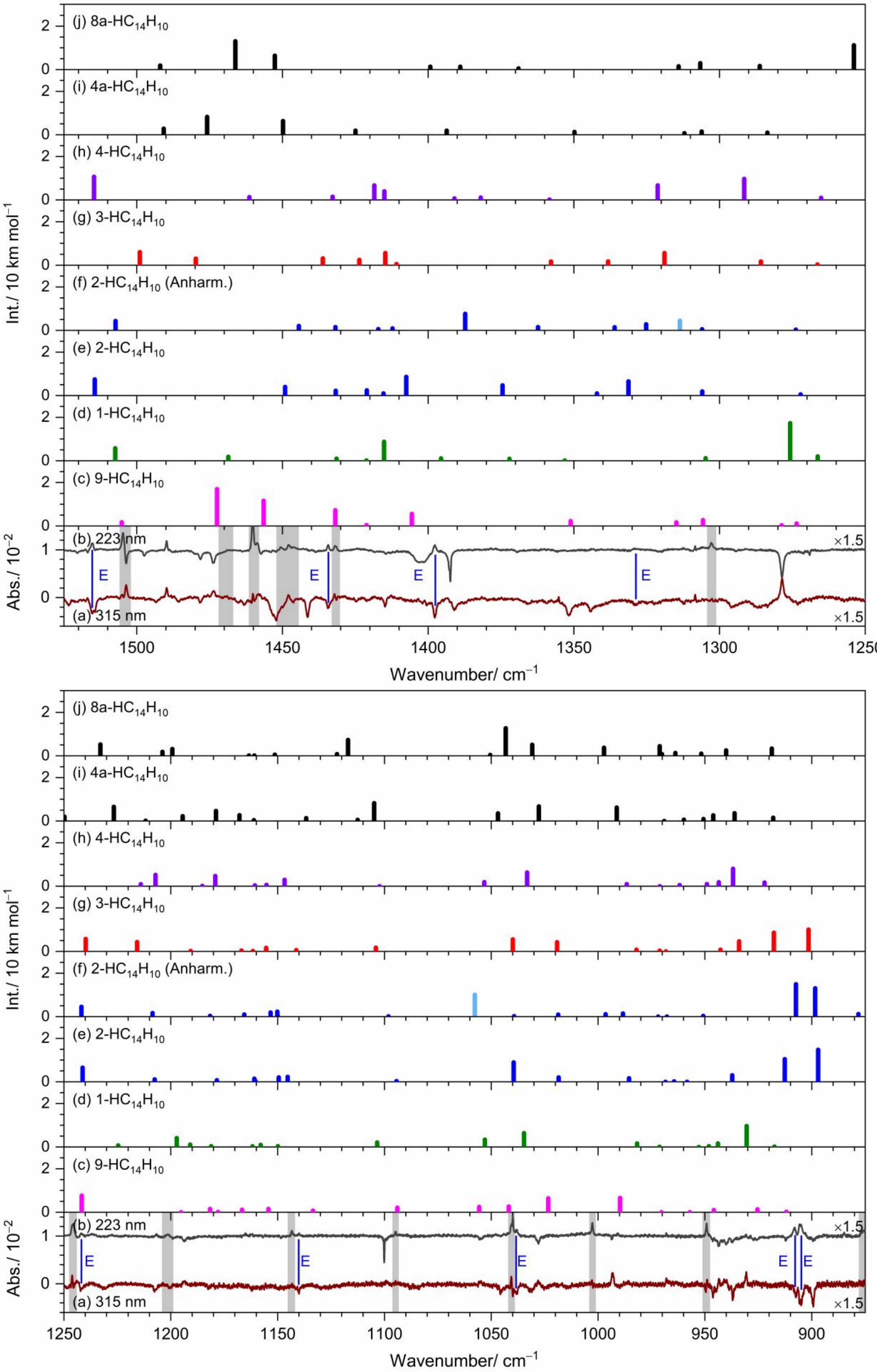

(j) 8a-HC14H10
(i) 4a-HC14H10
(h) 4-HC14H10
(g) 3-HC14H10
(f) 2-HC14H10 (Anharm.)
(e) 2-HC14H10
(d) 1-HC14H10
(c) 9-HC14H10
(b) 223 nm
(a) 315 nm
×1.5
E
Int./ 10 km mol−1
Abs./ 10−2
Wavenumber/ cm−1

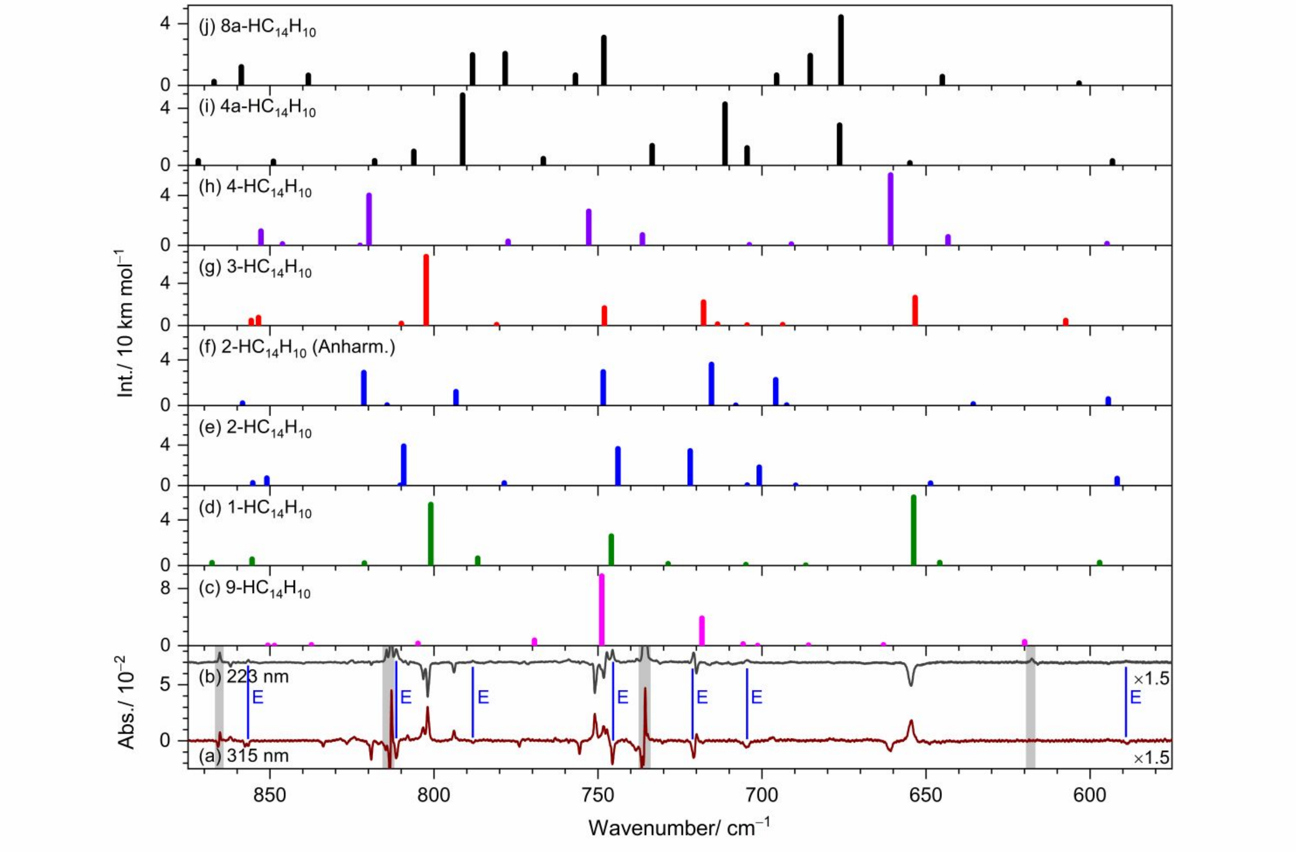

Figure S8. Comparison of lines in group E in the region 575−1525 cm^{-1} with stick spectra of seven isomers of $HC_{14}H_{10}$. Difference spectra after secondary irradiation at 315 nm (a) and 223 nm (b) are experimental spectra taken from Figure S2. Bands in group E are indicated with blue labels and lines. Spectral regions subjected to interference from the intense absorption of $C_{14}H_{10}$ are shaded gray. Baselines are shifted for clarity. The stick spectra (c)−(e), and (g)−(j) are based on scaled harmonic vibrational wavenumbers and IR intensities calculated with the B3LYP/6-311++G(d,p) method. The anharmonic vibrational stick spectrum of 2-$HC_{14}H_{10}$ is shown in trace (f), with combination and overtone bands in light color.

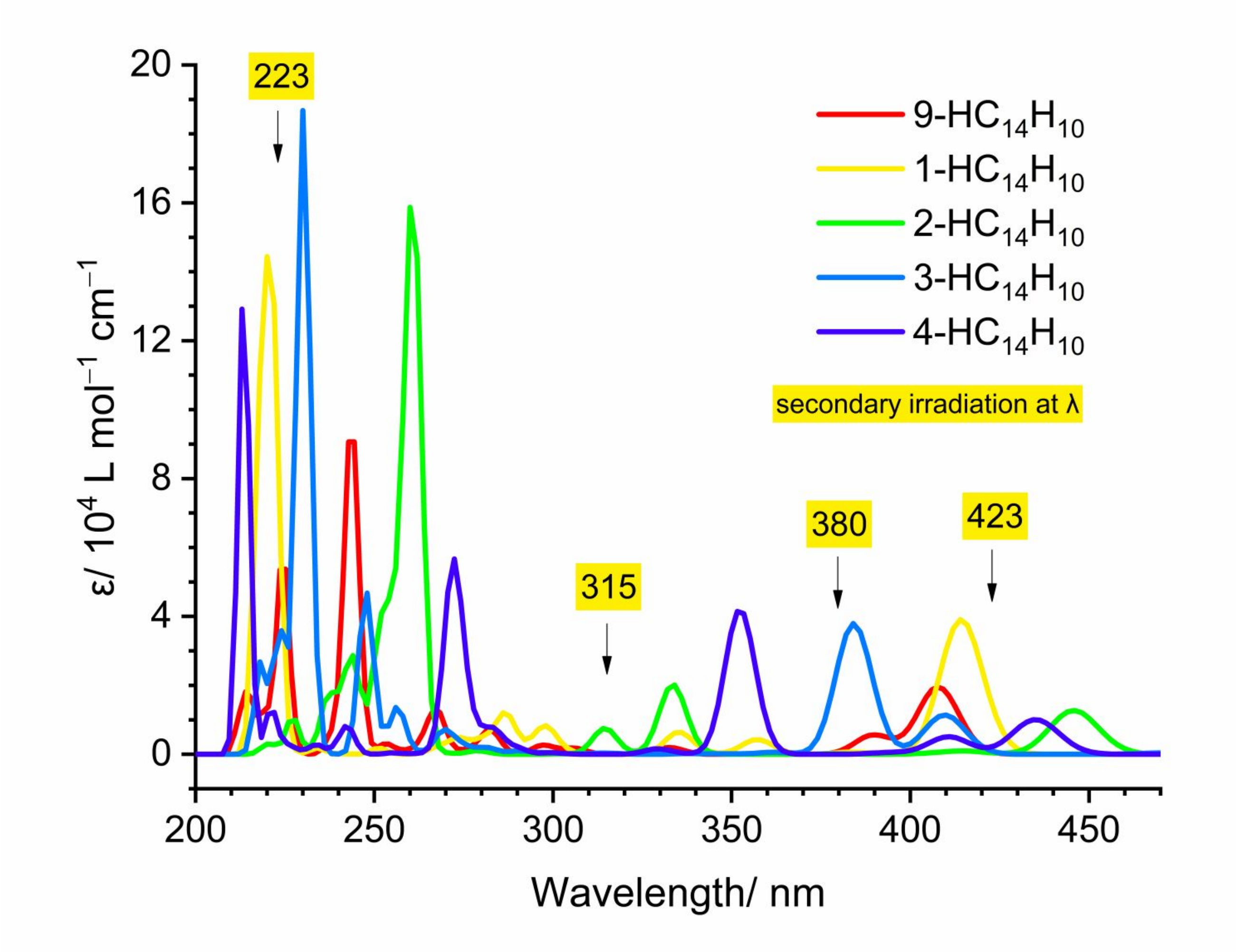

Figure S9. Calculated vertical electronic excitation spectra of 9-, 1-, 2-, 3-, and 4-$HC_{14}H_{10}$. The spectra are fitted with a Gaussian function with FWHM = 0.05 eV. The numbers in the yellow squares represent the selected wavelengths for secondary irradiation.
